# Barcode high-resolution melting (Bar-HRM) analysis to authenticate true cinnamon (*Cinnamomum verum*) from its adulterants and contaminants

**DOI:** 10.1371/journal.pone.0328808

**Published:** 2025-09-02

**Authors:** M. A. L. M. Peiris, Dhanesha Nanayakkara, Cristian Silva, Sachith P. Abeysundara, Priyanga Wijesinghe

**Affiliations:** 1 Postgraduate Institute of Science, University of Peradeniya, Peradeniya, Sri Lanka; 2 Department of Agricultural Biology, Faculty of Agriculture, University of Peradeniya, Peradeniya, Sri Lanka; 3 Department of Statistics and Computer Science, University of Peradeniya, Peradeniya, Sri Lanka,; 4 Department of Botany, Faculty of Science, University of Peradeniya, Peradeniya, Sri Lanka; Institute for Biological Research, University of Belgrade, SERBIA

## Abstract

Sri Lankan cinnamon, widely known as true cinnamon (*Cinnamomum verum*), is a world-renowned commodity. With the high market demand, many incidents have reported adulteration of true cinnamon with other cinnamon species such as *Cinnamomum aromaticum*, *Cinnamomum burmanni*, and *Cinnamomum loureiroi*. Moreover, the contamination of cinnamon products with fungi (*Aspergillus flavus*) has also significantly negatively impacted the cinnamon export market. Morphological and chemical detection of adulterations has limitations, benchmarking the necessity for precise and effective new detection methods. The current study reports gene-specific novel molecular markers that can be used in Barcode High-Resolution Melting (Bar-HRM) analysis to distinguish *C. verum* from other substitutes. Six barcode regions (*rbc*L, *trn*H-*psb*A, *mat*K, ITS2, *trn*L, *trn*L-*trn*F) were analyzed. The results demonstrate that *trn*H-*psb*A can effectively discriminate all selected cinnamon species from one another. Novel markers were designed to target the diagnostic nucleotide variations found within the designated barcode regions. Commercial cinnamon products and authentic samples of *C. verum* were used to validate the assay, and the DNA extraction protocol was optimized to ensure the acquisition of high-quality DNA. Bar-HRM was performed with the novel markers, and the four major cinnamon species in the international market were successfully distinguished. The spiked-in *A. flavus* DNA was also detected in a cinnamon admixture. Hence, these Bar-HRM conditions with the novel gene-specific markers can serve as an economical, efficient, and promising assay to detect the authenticity and purity of cinnamon samples.

## Introduction

The genus *Cinnamomum* belongs to the family Lauraceae, consisting of 110 species [1,2]. In the fifth century BC, *Cinnamomum verum* J. Presl; syn. *Cinnamomum zeylanicum* Blume became famous in Egypt and Europe. Large-scale commercial-level cultivations can be seen in Sri Lanka, India, Seychelles, Madagascar, Brazil, Southeast Asia, and other tropical countries [3]. In Sri Lanka, large-scale cultivations of *C. verum* are located along the coastal belt from Negombo to Matara, as well as in inland areas such as Kalutara, Ambalangoda, and Rathnapura [4].

The major constituent of *C. verum* leaf oil is eugenol, whereas its bark and roots predominantly contain cinnamaldehyde and camphor, respectively. In addition to these, linalool, cinnamyl acetate, benzyl benzoate, α-Pinene, α-Phellandrene, p-cymene, α-Humulene, and α-Terpineol can be found in all types of cinnamon oil in variable quantities, and the majority of these are bioactive [5]. These compounds have been found to possess anti-bacterial [6,7], anti-fungal [8–12], anti-viral [13,14], anti-diabetic [15,16], anti-oxidant [17], and anti-cancer [18,19] properties.

Four major types of cinnamon are currently available in the international market, *viz*., Ceylon cinnamon (*C. verum*), Chinese cinnamon [*C. aromaticum* Nees], Indonesian, Korintje, Java, or Padang cinnamon [*Cinnamomum burmanni* (Nees & T. Nees) Blume], and Vietnamese or Saigon cinnamon (*Cinnamomum loureiroi* Nees) with the latter three commonly referred to as cassia or cassia cinnamon [20–22]. Among these, only Ceylon cinnamon is recognized as the ‘true cinnamon’, and Sri Lanka is the premier exporter responsible for 80–90% of global production [22,23]. Cinnamon quills, quilling, powder, leaf oil, bark oil, and value-added products are significant exported cinnamon products. The cinnamon industry in Sri Lanka has encountered numerous challenges as a result of intense competition from inferior-quality substitutes [24], where some cassia products cost only a tenth of the price of *C. verum*, and this price difference has a significant impact on the elevated adulterations [25]. While cassia is more economical, it contains a substantial amount of the hepatotoxin, coumarine (up to 5%), whereas Ceylon cinnamon contains only trace amounts (about 0.004%) [26].

It has been reported that the *C. verum* bark and its products are frequently adulterated with *C. aromaticum* and *C. burmanni* for commercial purposes [26–28]. Nearly 51% of cinnamon products labeled as *C. verum* in the market were found to be *C. aromaticum*, with 10% being blend of *C. aromaticum* and *C. verum*, leaving only 39% that were truly *C. verum* [26]. In general, cassia is sold in the United States (US) as cinnamon, with the trade data showing that 90% of the cinnamon imported to the US was *C. burmanni* [29,30].

In addition to cinnamon adulterations, cinnamon products are particularly vulnerable to contamination, especially with *Aspergillus* and *Penicillium* fungal species. *Aspergillus terreus*, *Aspergillus glaucus*, *Aspergillus flavus*, *Aspergillus fumigatus*, *Aspergillus clavatus*, *Aspergillus niger*, and *Penicillium restrectum* are among the potential contaminating species [31,32]. Therefore, frequent testing of cinnamon products for contamination is crucial to ensure the safety of food, medicine, and an array of value-added products. In this study, we aimed to demonstrate the feasibility of detecting *A. flavus* contamination alongside cinnamon authentication.

The identification of plant species and their products primarily relies on morphological and chemical properties. However, in the case of cinnamon powder or admixtures of the products, morphological and chemical methods for detecting adulterants are ineffective due to their inherent limitations [25,33,34]. In this context, DNA-based identification methods such as barcoding provide a precise platform to identify the samples, even to the species level, based on the differences in their genetic makeup. Initially, the *mitochondrial cytochrome oxidase I* (*COI*) gene was utilized to identify various animal groups [35]. However, when researchers attempted to apply the *COI* region for plant identification, they did not achieve the anticipated results. Consequently, they explored different gene regions for identifying plant species, including coding regions (*rbc*L and *mat*K) and non-coding regions (*trn*L, *trn*L-*trn*F, *trn*T-*trn*L, and *trn*H-*psb*A) in the chloroplast genome, in addition to the internal transcribed spacers (ITS) in nuclear ribosomal DNA. These regions have been recommended for the identification and classification of plant species [36–38]. Furthermore, barcoding can be employed to detect fungal contamination in cinnamon products [39]. Nonetheless, DNA barcoding requires DNA sequencing facilities, with accessibility to these facilities being restricted in technology-limited countries [40], often resulting in a turnaround time of one month from sample collection to results. The new technique developed by combining DNA barcoding with high-resolution melting (HRM) analysis is known as Barcode high-resolution melting (Bar-HRM) analysis. Currently, this DNA Bar-HRM system has been successfully implemented to detect adulteration in food and herbal products [41–47]. Most importantly, Bar-HRM is a sequencing-free, close-tubed, precise, rapid, and economical analysis tool [42].

Here, we report the first application of Bar-HRM to detect adulterations and contaminations in cinnamon, along with the introduction of novel gene-specific Bar-HRM-compatible markers, selected through empirical testing of multiple primer pairs. Additionally, we used a modified protocol for DNA extraction from cinnamon bark, powder, and value-added products, enabling efficient DNA isolation. This assay efficiently distinguished among *C. aromaticum*, *C. burmanni*, C. *loureiroi,* and *C. verum*, as well as identifying contamination by A. *flavus*.

## Results and discussion

### *In-silico* studies and simulated HRM analysis

Based on the analyzed nucleotide sequences of the barcode regions *rbc*L, *trn*H-*psb*A, *mat*K, ITS2, *trn*L, and *trn*L-*trn*F of the four *Cinnamomum* species, Table 1 presents the calculated shortest and longest sequence lengths (in base pairs), characters used in analysis (base pairs), along with the percentage of conservative sites, variable sites, parsimony-informative sites, and singleton sites. The NCBI accession numbers of the retrieved sequences are given in the S1 Table in S1 File.

**Table 1:** Characteristics of the selected gene region derived through *in silico* analysis.

Property	Barcode region					
	*rbc*L	*trnH-psbA*	*mat*K	ITS2	*trn*L	*trn*L-*trn*F
**Number of deposited sequences in NCBI**						
***C. verum***	09	05	09	02	03	01
***C.aromaticum***	09	14	09	07	07	07
***C.burmanni***	07	11	09	05	02	01
***C.loureiroi***	01	01	01	01	00	00
**Shortest and longest sequence (bp)**	503-704	346-498	564-872	189-329	464-552	388-428
**Characters used in the analysis (bp)**	459	258	531	188	464	389
**Conservative site/total (%)**	442/459 (96.30)	245/258 (94.96)	516/531 (97.18)	140/193 (72.54)	461/464 (99.35)	388/389 (99.74)
**Variable site/total (%)**	17/459 (3.70)	13/258 (5.04)	15/531 (2.82)	52/193 (26.94)	3/464 (0.65)	1/389 (0.26)
**Parsimony-informative site/total (%)**	3/17 (17.65)	12/13 (92.31)	1/15 (6.67)	47/52 (90.38)	3/3 (100)	0/1 (0.00)
**Singleton site/total (%)**	14/17 (82.35)	1/13 (7.69)	14/15 (93.33)	5/52 (9.62)	0/1 (0.00)	1/1 (100)

The ITS2 region was found to exhibit the highest percentage of variable sites (26.94%), followed by the *trn*H-*psb*A (5.04%), *rbc*L (3.70%), and *mat*K (2.82%) regions. By contrast, the *trn*L (0.65%) and *trn*L-*trn*F (0.26%) regions exhibited the lowest percentages of variable sites. These findings indicated that, ITS2 region was the most suitable barcode region for species identification, whereas the *trn*L and *trn*L-*trn*F regions were demonstrated to be the least suitable. Details of the SNPs are provided in the S2 Table in S1 File.

**Fig 1:**
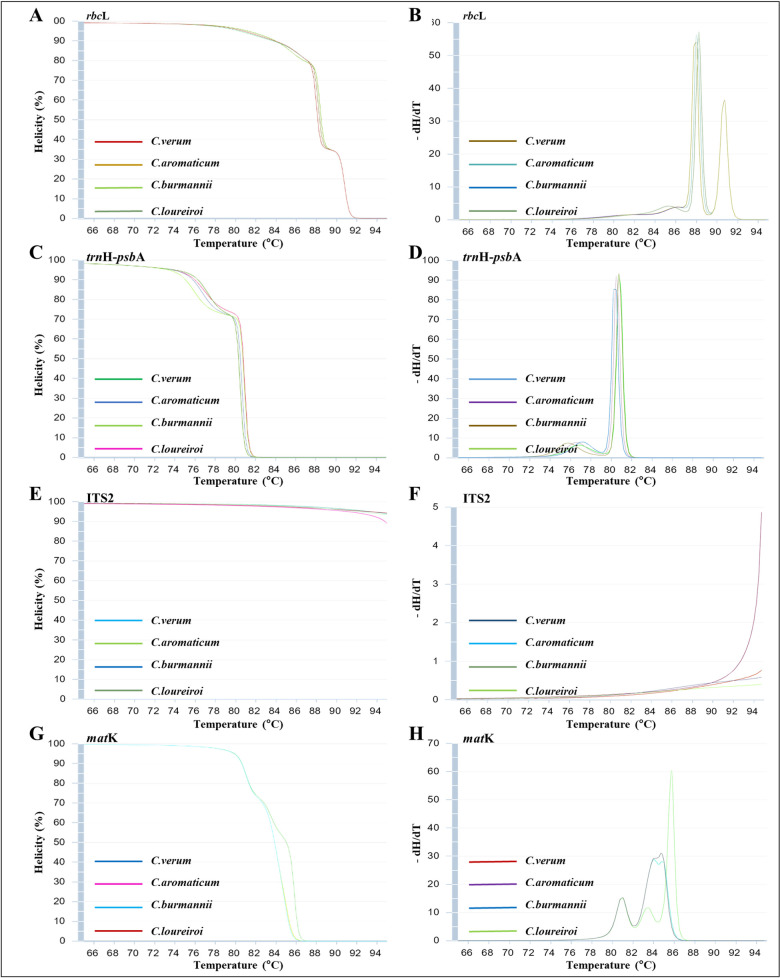
Normalized curves and melting temperature (*T*_m_) were generated from simulated HRM analyses (uMelt^SM^ v 2.4.1) (A) and (B) *rbc*L, (C) and (D) *trn*H-*psb*A, (E) and (F) ITS2, (G) and (H) *mat*K primers for the four cinnamon species: *C. verum*, *C. aromaticum*, *C. burmanni,* and *C*. *loureiroi.*

To further evaluate and confirm the suitability of barcode regions for use in Bar-HRM, four barcode regions, *rbc*L (459 bp), *trn*H-*psb*A (258 bp), *mat*K (531 bp), and ITS2 (188 bp), were subjected to a simulated HRM (uMelt^SM^ v 2.4.1) using the sequences that were meticulously trimmed at both the 5′ and 3′ ends to form a high-quality dataset for subsequent analysis. This analysis excluded *trn*L and *trn*L-*trn*F regions because of their extremely low percentages of variable sites. The results of the simulated HRM analysis provided the predicted melt curves for the selected barcode regions across four cinnamon species, demonstrating the ability of each barcode region to effectively differentiate *C. verum* from other cinnamon species (Fig 1).

Based on the normalized curves generated by uMelt^SM^ analysis, the barcode region *trn*H-*psb*A was the only region with adequate ability to discriminate all four selected cinnamon species from each other. By contrast, ITS2, the barcode region with the highest variable site percentage, could not distinguish *C. verum* from other cinnamon species based on its predicted melt curves. This limitation may arise because the ITS2 region often exhibits complex secondary structures and high GC content, which contribute to its thermodynamic stability. These characteristics can lead to intricate melting behaviors, complicating the differentiation of species [48]. Furthermore, while the ITS2 sequences exhibited a higher number of nucleotide variations compared to *trn*H-*psb*A, most of the variable sites within the *trn*H-*psb*A region are classified as parsimony-informative. Thus, interspecific variation of the ITS2 region is less effective than that of the *trn*H-*psb*A for the discrimination of Cinnamon species.

Therefore, *trn*H-*psb*A, the barcode region that exhibited the second highest percentage of variable sites among the regions assessed, was elected as the most suitable barcode for the design of novel gene-specific molecular markers in this study. This choice was not arbitrary but was guided by both the performance of our preliminary studies and the precedence set by previous studies on cinnamon and other plant taxa as described below. It is important to note that the discriminatory ability of DNA barcode regions can vary significantly across different plant lineages [43,44,49–51]. For example, a 149 bp fragment of the *rbc*L barcode region was employed to authenticate *Annona muricata* tea [46], and the *mat*K barcode region effectively differentiated between and identified *Hebanthe eriantha* and *Pfaffia glomerata*, commonly known as ‘Brazilian ginseng’ [52]. Furthermore, the *trn*H-*psb*A barcode region has also been utilized to identify *Uvaria* species [53], and has also demonstrated effectiveness in identifying various taxa, including species of the *Rhododendron* and *Physalis* genera, as well as species of the Apocynaceae and Fabaceae families [54–57]. This confirms the significant potential of the *trn*H-*psb*A region for species identification across diverse plant lineages because of its favorable characteristics, including high universality, reliable amplification efficiency, and proven effectiveness in species discrimination.

Leveraging the extensive applications of molecular barcoding, existing literature on *Cinnamomum* spp. highlights that one single-nucleotide polymorphism (SNP) within the *trn*L-*trn*F region and three sites within the *trn*L region have been used in molecular identification of *C. cassia*, *C. zeylanicum*, *C. burmanni*, and *C. sieboldii* [58]. Moreover, SNPs specific to *C. cassia* have been identified in the *rbc*L locus, enabling use of this locus to determine whether *C. cassia* was present as an adulterant in market samples of *C. verum* [30]. Furthermore, the ITS2 barcode region has been utilized for the identification of various *Cinnamomum* species, including *C. osmophloeum,* and for the construction of phylogenetic trees within the *Cinnamomum* genus [2,59,60]. In parallel, the authentication of *C. verum* has also been demonstrated using real-time quantitative PCR (qRT-PCR) assays [61]. It is essential to recognize here that qRT-PCR is primarily utilized for the real-time quantification of DNA during the amplification process. This technique offers quantitative data in the form of cycle threshold (C_t_) values, which enables the assessment of DNA copy numbers and is predominantly employed for gene expression analysis. The process typically consists of three stages: denaturation (generally conducted at 95°C), annealing, and extension (commonly at 70–72°C) [62]. In contrast, the Bar High-Resolution Melting (Bar-HRM) assay incorporates an additional melting phase, during which the instrument reheats the amplified product to generate comprehensive melting curve data. Moreover, Bar-HRM is specifically designed to differentiate and detect sequence variation, such as SNPs, by analyzing differences in melting temperatures (*T*_m_), which reflect the stability of DNA duplexes through the examination of post-amplification melting profiles. Importantly, the output generated by Bar-HRM signifies differences in sequence rather than DNA quantity, making it particularly effective for species-level identification. To our knowledge, the present study represents the first to use the Bar-HRM assay, along with the *trn*H-*psb*A barcode region, to effectively differentiate *C. verum* from its potential adulterants.

Weighing all, we derived the *trn*H-*psb*A barcode region both systematically as well as through trial and error as the most suitable region to distinguish *C. verum* from the three other most important cinnamon samples using Bar-HRM.

### DNA extraction and confirmation of the authenticity of cinnamon samples

In the international market, cinnamon is available in various forms, including dried bark, powder, mixtures, and value-added products. The initial step in any DNA-based methodology involves the extraction of high-quality DNA. Nonetheless, isolating DNA from dried bark presents greater challenges than from fresh materials. Cinnamon bark and powder typically contain higher amounts of polyphenols and polysaccharides than cinnamon leaves, which can act as PCR inhibitors, leading to erroneous interpretations. To minimize their presence, this protocol utilized a high concentration of Cetyl trimethylammonium bromide (CTAB) buffer (5%) to effectively remove polysaccharides [63,64]. Additionally, the effects of phenolic compounds were minimized using polyvinylpyrrolidone (PVP) [65,66]. The incorporation of β-mercaptoethanol delayed sample oxidation by phenolic compounds, facilitating DNA recovery [67], and sodium acetate was included to reduce DNA solubility in water, enhancing DNA precipitation [68,69]. These specific chemicals yielded high-quality DNA. Furthermore, this study introduces modifications to the standard protocol previously employed to isolate DNA from the barks of *Cinnamomum* species [70]; for instance, we tested the stirring speeds of 30, 70, 120, 160, and 170 rpm. Through a trial and error approach, the speed was reduced to 70 rpm to minimize the shearing and fragmentation of the DNA molecule, while the centrifugation speed was increased to 7000 rpm to enhance the precipitation of DNA, ensuring that the integrity and quality of the DNA remained intact [71].

DNA quantification was conducted with a nano spectrophotometer (Nabi UV/Vis, South Korea). The DNA concentration and quality control (QC) values were comparable to those obtained from leaf extraction, demonstrating the effectiveness of the modified DNA extraction protocol from the cinnamon bark.

PCR reactions were performed using the selected universal markers (Table 2) to amplify the target barcode regions from all cinnamon samples. The resulting PCR products were sequenced bi-directionally to validate the authenticity of the samples prior to further analysis. Based on BLAST homology search results, the species level of the selected cinnamon samples was confirmed. The sample information is provided in the S3 Table in S1 File.

**Table 2:** Universal and novel markers used for PCR amplification of DNA extracted from cinnamon samples.

Flanking gene	Forward primer (5′-3′)	Reverse primer (5′-3′)	Expected product size (bp)	Reference
***trn*H-*psb*A**	GTTATGCATGAACGTAATGCTC	CGCGCATGGTGGATTCACAAATC	500	Sang *et al*., 1997
***afl*R**	AACCGCATCCACAATCTCAT	AGTGCAGTTCGCTCAGAACA	798	Khare *et al*., 2018
***AP-trn*H*-psb*A**	GTTCCATCTACAAACGGATAATAC	GTCTTTATTACTTCACTCTCCTTCCT	368	This study

### Assaying of novel Bar-HRM markers in a standard PCR

To optimize and confirm the PCR program conditions and product amplification prior to Bar-HRM analysis, the newly designed Bar-HRM-compatible markers targeting the diagnostic SNP sites in the *trn*H-*psb*A barcode regions were assessed by standard PCR (Table 2). The marker *AP-trn*H*-psb*A resulted in a 368 bp amplicon without non-specific amplifications and primer dimers, thus validating the optimum PCR program conditions.

For the generation of a simulated HRM profile using the uMelt^SM^ software, a 258 bp region of the *trn*H-*psb*A (DNA positions 71–329, 5′–3′, NCBI reference sequence: MF137971.1) was employed to model the melting behavior. Subsequently, multiple primer pairs were designed to amplify the selected gene region, However, several of these primers did not yield the desired results, as indicated by low amplicon intensity, the presence of non-specific products, and significant primer dimer formation. Therefore, the primer pair used in the actual Bar-HRM assay was selected through a process of trial and error. This primer pair extended the amplified region by 100 bp, resulting in a 368 bp amplicon spanning positions 32–400 of the *trn*H-*psb*A region. The extension of the amplicon was intended to incorporate additional SNPs (S1B Fig in S1 File), As expected, the extended amplified region provided sufficient resolution to accurately differentiate all four cinnamon species with high accuracy, with the same graph pattern. Although the recommended amplicon length for HRM analysis typically ranges between 50 and 300 bp [42], considering thermodynamic properties, numerous studies have demonstrated that fragments extending to 300–400 bp can also be analyzed with sufficient sensitivity and specificity, yielding up to 100% accuracy [72–75] in similar context. Moreover, successful applications of amplicons longer than 300 bp have been reported for species-level identification [76]. Accordingly, the primer pair demonstrating the most robust and specific amplification was selected, ensuring clear and reliable PCR products suitable for high-resolution melting analysis.

### Assaying of *Aspergillus flavus*

*A. flavus* DNA was subjected to PCR amplification using the universal marker *afl*R, which targets the aflatoxin synthesis regulatory gene (*afl*R) (Table 2). Although an optimal PCR has been optimized for *afl*R amplification [39], the present study used the same PCR program and conditions used for the *Ap-trn*H-*psb*A marker of cinnamon to amplify the *afl*R gene. This protocol successfully obtained the amplicon in expected size for both cinnamon and *A. flavus* without any non-specific amplification. The compatibility of the PCR program and conditions for both the *Ap-trn*H-*psb*A and *afl*R markers enabled a single Bar-HRM assay to differentiation among all four cinnamon species, *C. verum, C. aromaticum*, *C. burmanni*, and *C*. *loureiroi*, while also detecting *A*. *flavus*.

### Bar-HRM analysis

Relatively early attempts to identify economically significant cinnamon species were based on their morphological characteristics and chemical compositions [77,78]. Subsequently, molecular techniques were incorporated for cinnamon species identification including Random Amplified Polymorphic DNA (RAPD) [33,79,80], Inter Simple Sequence Repeat (ISSR) [81,82], Sequence Related Amplified Polymorphism (SRAP) [33], and DNA barcoding analysis [2,30,58], all of which showed enhance precise identification of cinnamon species. The present study introduced a more advanced assay, Bar-HRM, which showed greater sensitivity than previous methods, making it highly effective in distinguishing among samples at the molecular level. This approach combined HRM with DNA barcoding, enabling rapid analysis of genetic variations in PCR amplicons [41]. During the HRM phase, the temperature was ramped from 65 °C to 95 °C. The DNA dye (SYTO® 9), intercalated within double-stranded DNA (dsDNA), was released during the dissociation process of dsDNA, resulting in a decrease in fluorescence intensity. Changes in fluorescence over time yielded a melt curve profile for the DNA sample with the melting temperature (*T*_m_) specific to each PCR product, providing a temperature-shifted melt curve for each sample, enabling discrimination at the species level [44].

**Fig 2:**
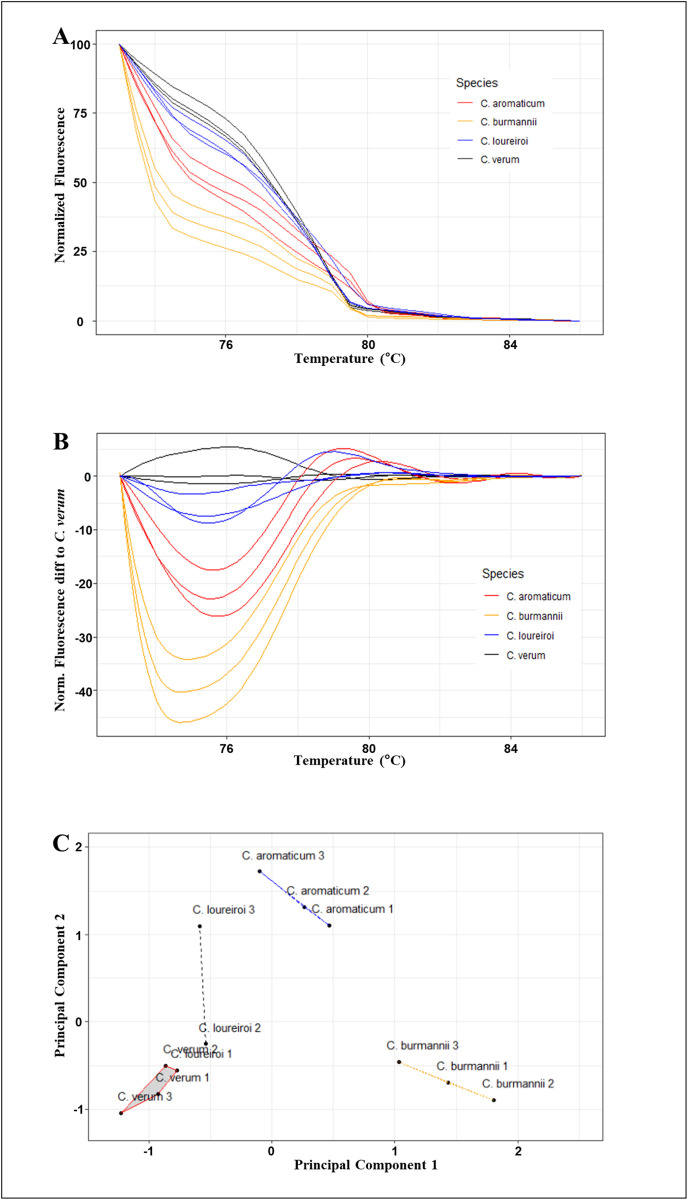
Melting curve profiles of amplicons obtained from the novel *AP*-*trn*H-*psb*A marker (A) Normalized melting curves, (B) Difference melting curves, and (C) Cluster plot showing the first two principal components and the four clusters for the *C. verum, C. aromaticum, C. burmanni,* and *C. loureiroi.*

The results of Bar-HRM analysis for the *trn*H-*psb*A barcode region in this study were presented as distinct melting curve profiles for each cinnamon species. These profiles were generated based on the patterns of normalized temperature-shifted curves and the corresponding difference plot. The barcode region *trn*H-*psb*A was selected for use in the Bar-HRM platform as it could discriminate all four selected cinnamon species, *C. verum*, *C. aromaticum, C. burmanni,* and *C. loureiroi,* from each other (Fig 2).

Bar-HRM analysis of the *trn*H-*psb*A region produced a normalized melting curve profile, characterized by a pre-melt region with a *T*_m_ ranging from 70.96°C to 71.4°C, and a post-melt region from 84.96°C to 86.36°C across all species (Fig 2A). This normalized melting curve pattern allowed for the successful differentiation of all cinnamon samples, achieving a confidence limit of 90%. Moreover, the distinct melting curve patterns showed greater resolution than the normalized melting curve profile in discriminating the four cinnamon samples, *C. verum*, *C. aromaticum, C. burmanni,* and *C. loureiroi* from each other (Fig 2B). The principal components obtained by Principal Component Analysis (PCA) were used to cluster and distinguish the *Cinnamomum* spp. based on Bar-HRM fluorescence data, with the four species were clearly distinguished by clustering into four clusters (Fig 2C). Nucleotide differences in the *trn*H-*psb*A barcode region were effective in distinguishing among the four species of cinnamon. Analysis of the 368 base pair fragments amplified by the *AP*-*trn*H-*psb*A primer pair revealed four specific SNPs for *C. verum*, seven for *C. aromaticum*, and one each for *C. burmanni* and *C. loureiroi*. Details of the SNPs are provided in the S2 Table in S1 File. These unique variations contributed to the observed differences in melting curve profiles, validating the efficacy of the selected barcode region for species authentication.

**Fig 3:**
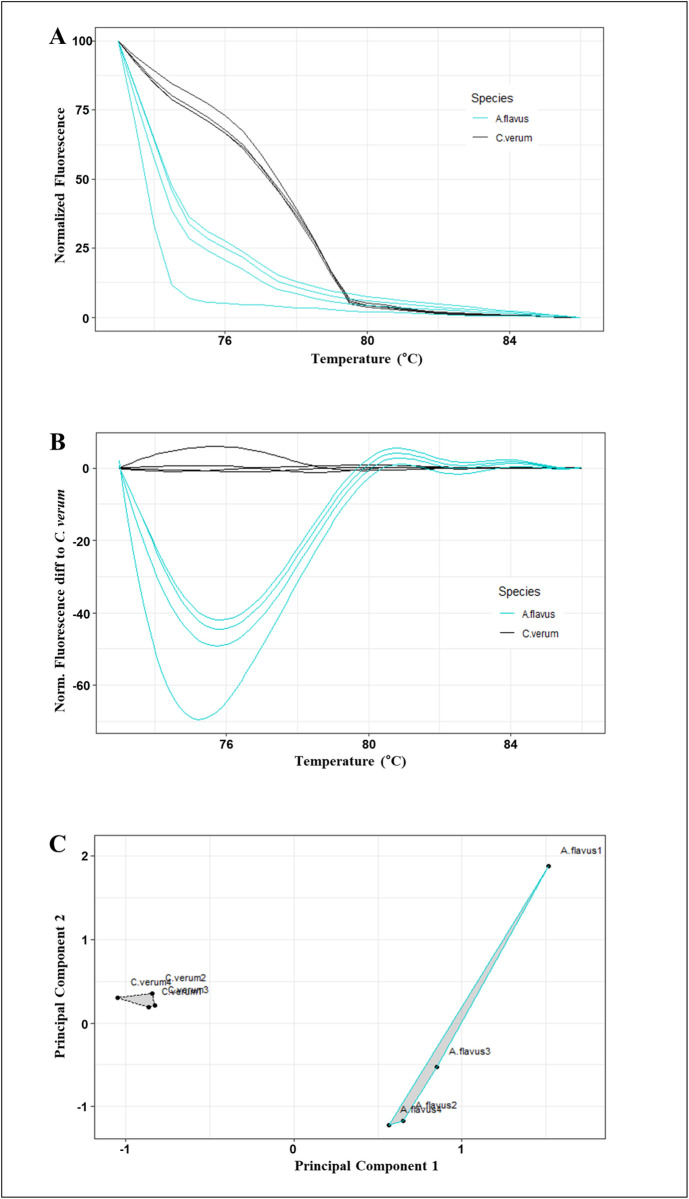
Melting curve profiles of amplicons obtained from the novel *AP*-*trn*H-*psb*A marker and *afl*R marker (multiplex) (A) Normalized melting curves, (B) Difference melting curves, and (C) Cluster plot drawn using the first two principal components and the two clusters for *C. verum* and *A. flavus.*

When developing an assay to detect both cinnamon adulterants and fungal contamination from *A. flavus* in cinnamon products, it is essential to amplify both cinnamon-specific primers (*AP-*trn**H-*psb*A) and fungus-specific primers (*afl*R) within the same PCR mixture under the same PCR conditions. The PCR conditions should be optimized to avoid the generation of non-specific bands and primer dimers. These nonspecific bands could obscure the melting behavior of the target amplicon, making it difficult to distinguish between specific and nonspecific products [83,84]. Bar-HRM analysis of the *trn*H-*psb*A regions of all four cinnamon species and the *afl*R region of *A. flavus* yielded a normalized melting curve profile with a pre-melt region of *T*_m_ from 70.96°C to 71.4°C and a post-melt region from 84.96°C to 86.36°C (Fig 3). A similar assay was conducted with all four cinnamon species, which showed a distinct separation of profiles. The data are illustrated in the S2 Fig in S1 File.

The melting curve patterns generated for *C. verum* and *A. flavus* were distinct, demonstrating the ability of the multiplex to distinguish between *C. verum* and *A. flavus* (Figs 3A and B). Based on Bar-HRM fluorescence data, the first two principal components derived from the PCA could distinguish *C. verum* from *A. flavu*s, with the two species clearly forming two separate clusters (Fig 3C).

**Fig 4:**
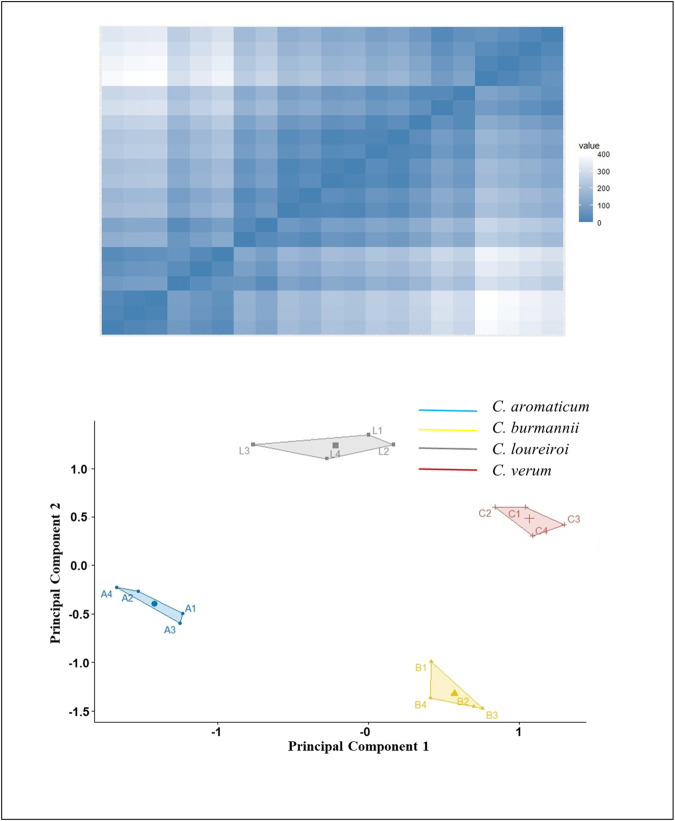
The cluster tendency of Bar-HRM fluorescence data obtained from the novel *AP*-*trn*H-*psb*A marker was assessed by Hopkins’s statistics, and the K-means algorithm demonstrated four separate clusters of the *C. verum, C. aromaticum, C. burmanni,* and *C. loureiroi.*

The clustering tendency of the melting curves was evaluated using Hopkins’s statistics. The PCA revealed that first, three principal components accounted for 99% of the total variability, thereby facilitating subsequent cluster analysis. K-means clustering indicated a clear distinction among the four species of cinnamon, showing effective separation without misclustering. The resulting cluster plot (Fig 4) closely resembles Fig 2C, further confirming the ability of this method to detect adulterations in cinnamon.

To validate the practical application of our method, we analyzed six independent commercial cinnamon products (S4 Fig in S1 File). Out of these, four products were identified as *C. verum* (Test samples 1, 2, 3, and 4), while two samples were classified as cassia cinnamon (Test samples 5 and 6).

This method also has potential in the development of a classification model for the accurate identification of the four main cinnamon species based on HRM curve analysis.

**Fig 5:**
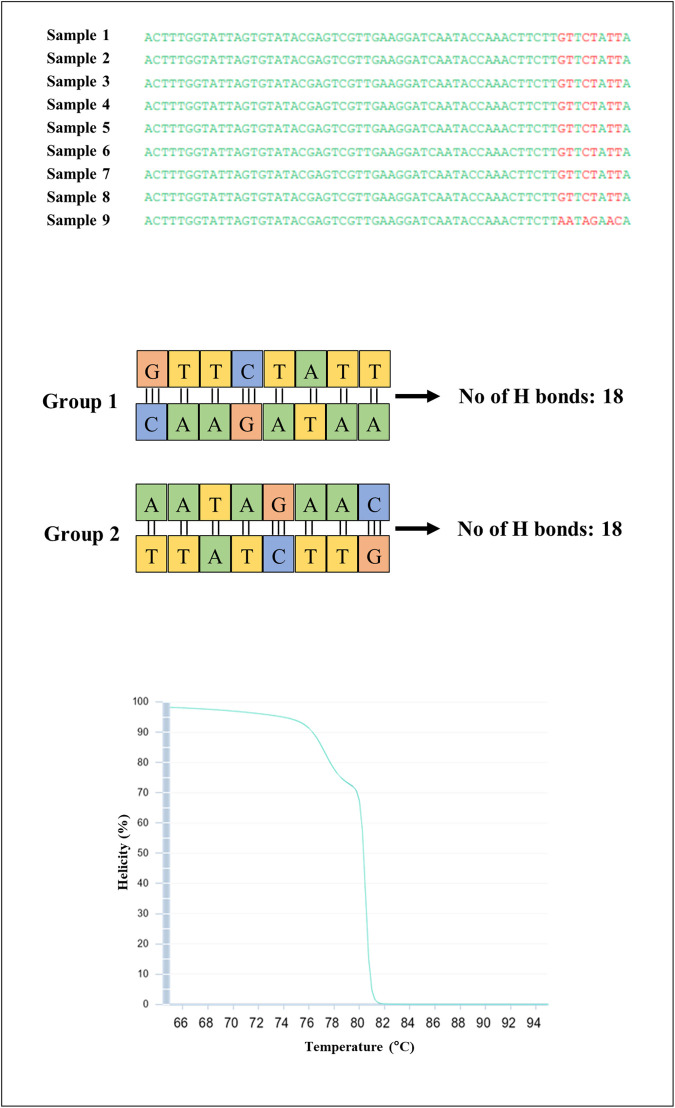
Sequence alignment results of collected samples for the *trn*H-*psb*A region, focusing on H bond comparison, and a normalized curve was generated from simulated HRM analyses (uMelt^SM^ v 2.4.1) for the two groups of *C. verum.*

The primary objective of this study was to develop a method that could distinguish Ceylon cinnamon from the cassia samples. Because the biology of the *C. verum* flowering pattern can promote genetic diversity due to the phenomenon known as protogynous dichogamy [85], the stability and suitability of the SNPs for the Bar-HRM assay were assessed in various collected cinnamon samples. Fresh leaf samples were collected from five distinct agricultural fields in Sri Lanka (Ambalangoda, Southern Province, Sri Lanka) where cinnamon is cultivated to export. The samples were subjected to Sanger sequencing and the resulting sequences were subsequently deposited in the National Center for Biotechnology Information (NCBI) database (PQ348605, PQ348606, PQ348607, PQ348608, PQ348609, PQ348610, PQ348611, PQ348612, PQ348613, PQ348614, PQ348615). Following this, an analysis of the sequences was conducted to identify the SNPs. Based on the alignment file, we identified two distinct groups of *C. verum*. One group featured 5’-GTTCTATT’ while the other contained 5’-AATAGAAC’. These groups were a result of chromosomal rearrangements, identified as inversion mutation. This nucleotide variation is observed within positions 86–93 of the *trn*H-*psb*A region of *C. verum* [NCBI reference sequence: AF268784.1]. To further validate this nucleotide variation, relevant sequences deposited to date in the NCBI were evaluated. The alignment files are incorporated within S3 Fig in S1 File. The results indicated that all sequences exhibited either one of the nucleotide variations as those previously identified in *C. verum,* thereby reinforcing the applicability of the assay regardless of the cinnamon collection site (Fig 5). Because the developed assay was based primarily on the melting temperatures of a specific genetic region, however, the number of hydrogen (H) bonds in that region significantly affects the shape of the melting curves. As the chromosomal rearrangement that contributed to the origin of the two cinnamon types belongs to an ‘inversion’, both regions exhibited 18 H bonds, with a balanced ratio giving identical melting signatures, benchmarking the feasibility of the assay across all cinnamon samples available. This balance was further supported by the simulated HRM analyses without any separation (Fig 5) using uMelt^SM^ v 2.4.1. Consequently, these results indicate that the developed assay was not affected by the protogynous dichogamy exhibited by the flowers of cinnamon and can effectively distinguish among *C. verum, C. aromaticum, C. burmanni,* and *C. loureiroi*.

This study has successfully demonstrated the effectiveness of the designed assay to accurately identify four main species of cinnamon. The next step of this study will focus on precisely quantifying the percentage of adulteration in an admixture. The high sensitivity of Bar-HRM technology allows for the detection of even minute levels (as low as 1%) of adulteration through distinct shifts in the melting curve profile [86,87]. This level of sensitivity has been widely supported in previous literature, which demonstrates the capacity of HRM to reliably detect and differentiate plant species and identify adulterants in admixtures [41,88–90]. The present findings, however, developed a robust and flexible screening method coupling the cinnamon biology with the power of Bar-HRM, providing valuable insights into the precise identification of the four main species of cinnamon in the world and of *A. flavus* contamination in cinnamon.

## Conclusion

This study presents a novel application of DNA barcoding coupled with high-resolution melting analysis (Bar-HRM) for the authentication of cinnamon species, specifically aimed at distinguishing *C. verum* from its common adulterants. Targeting the *trn*H-*psbA* intergenic spacer, we have developed a rapid, cost-effective, and robust assay suitable for processed cinnamon products, where conventional morphological and chemical identification methods are often limited.

Our findings demonstrate that the Bar-HRM approach can effectively differentiate *C. verum* from closely related cassia species, including *C. aromaticum, C. burmanni, and C. loureiroi*, thereby ensuring the authenticity of true cinnamon in commercial products. Furthermore, we introduce a novel assay based on the universal *afl*R marker, which facilitates the detection of *A. flavus* contamination. This advancement enables the simultaneous detection of adulteration and potential mycotoxin-producing fungi in cinnamon samples. Grounded in the principles of advanced cinnamon biology and molecular diagnostics, this innovative dual-function assay offers a timely and practical solution for ensuring the authenticity and safety of cinnamon in the global spice trade.

## Materials and methods

### Sequence retrieval, alignment, and *in-silico* analysis

DNA barcode sequences of *C. verum*, *C. aromaticum*, *C. burmanni,* and *C*. *loureiroi* were retrieved from NCBI repository (https://www.ncbi.nlm.nih.gov/) using the keyword “*Cinnamomum”* and barcode region viz., *rbc*L, *mat*K, *trn*L, *trn*L-*trn*F, *trn*H-*psb*A, ITS1, ITS2, and *trn*T-*trn*L. Retrieved sequences were 5’ and 3’ trimmed [documented under the name of characters used in the analysis (bp) in Table 1], and aligned and analyzed in MEGA v7.0 [91]. To determine the suitable barcode regions that can be applied in Bar-HRM, parameters such as conserved site (%), variable site (%), parsimony-informative site (%), singleton site (%), and the number of specific SNPs were calculated using MEGA v7.0 software.

### Simulated high-resolution melting (HRM) analysis and development of novel markers for Bar-HRM

Barcode regions were then used to perform the simulated HRM using the uMelt^SM^ v 2.4.1 application [92] to predict the melting profile for each selected barcode region to test the feasibility for Bar-HRM [46]. Barcode regions that can be used to discriminate cinnamon species were selected based on the simulated HRM analysis, and specific novel markers for Bar-HRM were manually designed, targeting the identified diagnostic SNP sites. When designing the markers, 5’ to 3’ DNA strand was used and additionally, two main criteria were considered to obtain successful results in the HRM analysis: (i) the primer pair should generate a PCR product not exceeding 400 bp [73–75], (ii) the primer pairs should cover enough variable sites to enable discrimination among the tested species. Furthermore, primer properties (GC clamps, self-dimer formation, hairpin loop formation, and cross-dimer formation) were analyzed using NetPrimer software (http://www.premierbiosoft.com/netprimer) to evaluate the suitability of the designed primers for the Bar-HRM analysis.

### Sample collection

Fresh cinnamon leaf samples were collected from five distinct cultivated fields in Ambalangoda, Southern Province, Sri Lanka, where cinnamon is cultivated for the export market, and the C. *verum* was authenticated with the consent of the respective farmers. We didn’t collect any wild samples. Consequently, obtaining permission or licenses for sample collection was not required. The collected samples were identified using general morphological characteristics by the research team. The collected specimens were meticulously cross-referenced with the voucher specimens at the National Herbarium (PDA) at the Royal Botanic Garden, Peradeniya, Sri Lanka, to ensure accuracy*.* Five types of *C. verum* products, including quills and powders, were sourced from local markets. *C. aromaticum*, *C. burmanni*, and *C*. *loureiroi* samples were purchased from the United States and the Republic of South Korea. The authenticity of the samples was confirmed by Sanger sequencing. The pure fungal culture of *A. flavus* was obtained from the Tea Research Institute of Sri Lanka, Thalawakelle, Sri Lanka.

### DNA extraction from cinnamon samples

#### Cinnamon leaves.

DNA extraction was performed following a CTAB method [93] using approximately 1 g of healthy cinnamon leaves from each sample. Leaf samples were separately ground into a fine powder and incubated at 60 ⁰C for 30 minutes in 1.5 ml of preheated (60 ⁰C for 15 minutes) 2% Cetyl trimethylammonium bromide (CTAB) extraction buffer [1M Tris base, 0.5 M ethylenediaminetetraacetic acid (EDTA), 5 M sodium chloride (NaCl), 0.2% β-mercaptoethanol]. Then, the samples were cooled down to room temperature, and 2/3 volume of chloroform: isoamyl alcohol (24:1, v/v) was added. Samples were inverted several times and centrifuged at 12,000 rpm for 2 min. The aqueous phase was transferred to a new tube, and the chloroform: isoamyl alcohol (24:1, v/v) step was repeated. Then 2/3 volume of ice-cold isopropanol was added, the tube inverted several times, and stored overnight at – 20 ⁰C. The tubes were centrifuged at 12,000 rpm for 10 minutes. The pellet was washed with ice-cold 70% ethanol, dried, and dissolved in nuclease-free water. The samples were quantified using a nano spectrophotometer (Nabi UV/Vis, South Korea).

#### Cinnamon bark and powder.

Herein, we report a modified protocol for DNA extraction from cinnamon bark and cinnamon powder that is optimized based on the protocol in Swetha *et al*., 2014 [70]. Approximately 1 g of each sample was measured and homogenized using 10 mL of preheated 5% CTAB extraction buffer (1 M Tris base, 0.5 M EDTA, 3 M NaCl, 5% CTAB, 1% polyvinylpyrrolidone (PVP), and 0.3% β-mercaptoethanol). Then, the homogenized samples were incubated at 65 °C in a shaking water bath with constant stirring at 70 rpm for 2 h. An equal volume of chloroform: isoamyl alcohol (24:1, v/v) was added after the samples were cooled down to room temperature. Samples were centrifuged at 3500 rpm for 15 min. The supernatant was transferred to a new tube. One-third of the sample volume of 3 M Sodium acetate (pH 5.2) and 2/3 of the sample volume of chloroform: isoamyl alcohol (24:1, v/v) were added to the supernatant. The samples were again centrifuged for 15 min at 3500 rpm, and the resulting supernatant was separated. An equal volume of ice-cold isopropanol was added and kept overnight at – 40 °C. Incubated samples were centrifuged at 7000 rpm for 20 min, and the resulting pellet was washed with 70% ethanol. The pellet was dried and dissolved in nuclease-free water. The samples were quantified using the nano spectrophotometer (Nabi UV/Vis, South Korea).

### Preparation of *A. flavus* for PCR amplification

The authentic cultures were sub-cultured in potato dextrose agar (PDA) at room temperature using the streak plate technique. The direct colony polymerase chain reaction (DCPCR) method was performed on the mycelia grown on the PDA plates. Briefly, a loop of fungal mycelium was added to a sterilized 500 µL of nuclease-free water sample and mixed. A series of thermal shocks was given as 10s, 10s, 20s, and 10s using a microwave oven. Then, the sample was vortexed vigorously for 3–5 minutes following a centrifugation step at 8000 rpm for 10 minutes. The resulting aliquot was used as the PCR template.

### PCR amplification and sequencing

The PCR was performed to amplify the barcode regions *trn*H-*psb*A and *afl*R using three gene-specific universal markers (Table 2). The total volume of the PCR reaction mixture was 10 µL which includes 24 ng/µl template DNA, 0.2 µM of forward and reverse primer (IDT, USA), 1 × Colorless Go Taq® Flexi buffer (Promega, USA), 1 U Go Taq® DNA polymerase (Promega, USA), 1.5 mM MgCl_2_ (Promega, USA), 0.1 mM dNTPs (Bio Basic Inc., Canada). The template DNA was replaced with nuclease-free water in the no-template control. The PCR was performed in a thermal cycler (Takara, USA) following different PCR programs for the selected genes. For *trn*H-*psb*A and *afl*R consisted of an initial denaturation at 92°C for 1 min, denaturation at 94°C for 1 min, annealing at 52°C for 1 min, extension at 64°C for 1 min, final extension at 64°C for 8 min with 35 cycles.

The SNPs of all resulting amplified products of cinnamon were confirmed by bi-directional Sanger sequencing using Applied Biosystems Genetic Analyzer 3500 in the Department of Molecular Biology and Biotechnology, Faculty of Science, University of Peradeniya, Sri Lanka. Minor changes in sequences were manually checked and edited using Bioedit software (v7.0.5.3) by referring to respective chromatograms (Chromas v2.6.6). All these sequences were subjected to a homology search against the NCBI database, and confirmation of the authenticity of the selected cinnamon samples was done based on sequence similarity.

### Bar-HRM analysis

Real-time PCR was performed to distinguish the four cinnamon species using the newly designed gene-specific diagnostic primer pairs. The final PCR reaction volume was 10 µL which includes 24 ng/µl template DNA, 0.2 µM of forward and reverse primers (IDT, USA), 2 µM of SYTO® 9 green-fluorescent nucleic acid stain (Invitrogen, USA), 1 × Colorless Go Taq® Flexi buffer (Promega, USA), 1 U Go Taq® DNA polymerase (Promega, USA), 1.5 mM MgCl_2_ (Promega, USA), and 0.1 mM dNTPs (Bio Basic Inc., Canada) in a Rotor-Gene Q thermal cycler using Q-Rex v1.0.0 plugin (Qiagen, Germany). The template DNA was replaced with nuclease-free water in the no-template control. Different PCR programs were used for the amplification. For *trn*H-*psb*A and *afl*R; an initial denaturation at 92 °C for 1 min, denaturation at 94 °C for 1 min, annealing at 52°C for 1 min, extension at 64 °C for 1 min, repeated for 35 cycles, and a final extension at 64 °C for 8 min followed by a melt curve analysis at 65 ⁰C to 95 ⁰C with a ramping rate of 0.05 ⁰C. The Rotor-Gene Q Series Software version 1.0.0 was used to analyze the captured fluorescence signals. A normalized curve of declining fluorescence with escalating temperature was plotted. *T*_*m*_ was given by the negative derivative of fluorescence (F) over the temperature (T) curve (dF/dT). Pre- and post-melt normalization regions were set to express the main temperature boundaries of the normalized and different schemes, where the distinct melting profile of each species is detected to derive normalized melting curve profiles. The melting curves were analyzed using the Rotor-Gene Q Series Software version 1.0.0 to distinguish *Cinnamon* species*.* The means of conventional derivative plots showed the melting temperature (*T*_*m*_) value for *trn*H-*psb*A amplicons. All selected cinnamon samples were triplicated, and three independent Bar-HRM runs were carried out to check the reproducibility and confirm the results.

### Statistical analysis

Sixteen normalized melting curves from four different cinnamon species were analyzed using R statistical software 4.2.1 [94]. To have a maximum separation between clusters and to minimize miscalculations, the temperature range between 70⁰C to 90⁰C was selected for subsequent analysis. This range was manually selected after a thorough examination of the raw fluorescence data against the temperature plot [95]. The chosen interval encompasses both the pre-melt region (70.96 °C to 71.4 °C) and the post-melt region (84.96 °C to 86.36 °C), thereby effectively illustrating the distinctions and separations among the species under investigation. Hopkin statistics were estimated by the factoextra R package [96] to determine the tendency of clustering of the normalized HRM dataset derived from the Rotor-Gene Q Series Software version 1.0.0.

In this software, the process of melting curve normalization is accomplished through the delineation of two key regions: the pre-melt region, which denotes baseline fluorescence prior to the denaturation of DNA, and the post-melt region, which indicates fluorescence following the complete melting of DNA. Fluorescence data within these specified regions undergo normalization via a scaling method, whereby pre-melt fluorescence is standardized to 100% and post-melt fluorescence is scaled to 0%. This normalization process is carried out by fitting a line of best fit, with the highest recorded fluorescence values assigned a value of 100 and the lowest values set to 0. The fluorescence average and slope observed within the transitional zone between the pre- and post-melt regions are utilized in the normalization calculations. Consequently, normalized melt curves are produced for comparative analysis across various samples. Moreover, the software enables the visualization of samples as difference plots, with respect to a selected control, thereby enhancing the ability to detect subtle sequence variations with greater precision. [97]. Linear dimension reduction was done by Principal Component Analysis (PCA), and the optimal number of principal components was identified [98]. K- means algorithm was used to cluster the fluorescence data. The optimal number of clusters was identified using standard methods such as the elbow, silhouette, and Gap statistics [99].

## Supporting information

S1 File Supplementary data. **S1 Fig.** Alignment of sequences retrieved from GenBank (NCBI) for *trn*H-*psb*A barcode region (A) Gene region that was used in simulated HRM profile using the uMeltSM software, (B) Gene region that was used in the actual Bar-HRM assay. **S2 Fig.** Melting curve profiles of amplicons obtained from the novel *AP*-*trn*H-*psb*A marker and *afl*R marker (multiplex) (A) Normalized melting curves, (B) Difference melting curves of the *C. verum*, *C. aromaticum*, *C. burmanni*, *C. loureiroi*, and *A. flavus*. **S3 Fig.** Sequence alignments of *C. verum* in the *trn*H-*psb*A barcode region(A) Sequences of samples collected from agricultural fields and (B) *C. verum* sequences retrieved from GenBank (NCBI). **S4 Fig.** Difference melting curves of the *C. verum*, *C. aromaticum*, *C. burmanni*, *C. loureiroi*, and commercial products were derived from amplicons obtained using the novel *AP*-*trn*H-*psb*A marker. **S1 Table.** DNA sequences corresponding to the *rbc*L, *trn*H-*psb*A, *mat*K, ITS2, *trn*L, and *trn*L-*trn*F regions of *Cinnamomum* species were retrieved from GenBank (NCBI), with accession numbers documented for each species. **S2 Table.** Comparison of nucleotide variation among Cinnamomum species (A) Number of SNPs specific to each gene region which can be used to distinguish up to species level, (B) position of nucleotide variation of *rbc*L, and *trn*H-*psb*A, *trn*L, *trn*L-*trn*F *mat*K, and ITS2 regions of *Cinnamomum* species. **S3 Table.** Details of the samples that were used for the study.
(DOCX)
