# Supplementary material for: Barcode high-resolution melting (Bar-HRM) analysis to authenticate true cinnamon (Cinnamomum verum) from its adulterants and contaminants
Source: PLoS One. 2025 Sep 2;20(9):e0328808. doi: 10.1371/journal.pone.0328808 (PMC12404453; doi:10.1371/journal.pone.0328808)
Supplement: S1 File — Supplementary data. S1 Fig. Alignment of sequences retrieved from GenBank (NCBI) for trnH-psbA barcode region (A) Gene region that was used in simulated HRM profile using the uMeltSM software, (B) Gene region that was used in the actual Bar-HRM assay. S2 Fig. Melting curve profiles of amplicons obtained from the novel AP-trnH-psbA marker and aflR marker (multiplex) (A) Normalized melting curves, (B) Difference melting curves of the C. verum, C. aromaticum, C. burmanni, C. loureiroi, and A. flavus. S3 Fig. Sequence alignments of C. verum in the trnH-psbA barcode region(A) Sequences of samples collected from agricultural fields and (B) C. verum sequences retrieved from GenBank (NCBI). S4 Fig. Difference melting curves of the C. verum, C. aromaticum, C. burmanni, C. loureiroi, and commercial products were derived from amplicons obtained using the novel AP-trnH-psbA marker. S1 Table. DNA sequences corresponding to the rbcL, trnH-psbA, matK, ITS2, trnL, and trnL-trnF regions of Cinnamomum species were retrieved from GenBank (NCBI), with accession numbers documented for each species. S2 Table. Comparison of nucleotide variation among Cinnamomum species (A) Number of SNPs specific to each gene region which can be used to distinguish up to species level, (B) position of nucleotide variation of rbcL, and trnH-psbA, trnL, trnL-trnF matK, and ITS2 regions of Cinnamomum species. S3 Table. Details of the samples that were used for the study. (DOCX) [file pone.0328808.s001.docx]

**Barcode High-Resolution Melting (Bar-HRM) analysis to authenticate true cinnamon (*Cinnamomum verum*) from its adulterants and contaminants**

M. A. L. M. Peiris^1^, Dhanesha Nanayakkara^3^, Cristian Silva^1^, Sachith P. Abeysundara^4^ and Priyanga Wijesinghe^1,2*^

^1^Postgraduate Institute of Science, University of Peradeniya, Peradeniya, Sri Lanka

^2^Department of Botany, Faculty of Science, University of Peradeniya, Peradeniya, Sri Lanka,

^3^ Department of Agricultural Biology, Faculty of Agriculture, University of Peradeniya, Peradeniya, Sri Lanka

^4^Department of Statistics and Computer Science, University of Peradeniya, Peradeniya, Sri Lanka

* Corresponding author

E-mail: [priyangaw@sci.pdn.ac.lk](mailto:priyangaw@sci.pdn.ac.lk) (PW)

**S1 Fig.** **Alignment of sequences retrieved from GenBank (NCBI) for *trn*H*-psb*A barcode region** (A) Gene region that was used in simulated HRM profile using the uMelt^SM^ software, (B) Gene region that was used in the actual Bar-HRM assay.

**A**

C.aromaticumHM019388.1 TCGTTGAAGGATCAATACCAAACTTCTTAATAGAACAAGAAGTTTGGTATTGATCTATTT C.aromaticumHM019389.1 TCGTTGAAGGATCAATACCAAACTTCTTAATAGAACAAGAAGTTTGGTATTGATCTATTT C.aromaticumKF978096.1 TCGTTGAAGGATCAATACCAAACTTCTTAATAGAACAAGAAGTTTGGTATTGATCTATTT C.aromaticumKF978097.1 TCGTTGAAGGATCAATACCAAACTTCTTAATAGAACAAGAAGTTTGGTATTGATCTATTT C.aromaticumKF978098.1 TCGTTGAAGGATCAATACCAAACTTCTTAATAGAACAAGAAGTTTGGTATTGATCTATTT C.aromaticumKF978099.1 TCGTTGAAGGATCAATACCAAACTTCTTAATAGAACAAGAAGTTTGGTATTGATCTATTT C.aromaticumKX675171.1 TCGTTGAAGGATCAATACCAAACTTCTTAATAGAACAAGAAGTTTGGTATTGATCTATTT C.aromaticumKX675172.1 TCGTTGAAGGATCAATACCAAACTTCTTAATAGAACAAGAAGTTTGGTATTGATCTATTT C.aromaticumKX675173.1 TCGTTGAAGGATCAATACCAAACTTCTTAATAGAACAAGAAGTTTGGTATTGATCTATTT C.aromaticumMF096909.1 TCGTTGAAGGATCAATACCAAACTTCTTAATAGAACAAGAAGTTTGGTATTGATCTATTT C.aromaticumMF096910.1 TCGTTGAAGGATCAATACCAAACTTCTTAATAGAACAAGAAGTTTGGTATTGATCTATTT C.aromaticumMF096911.1 TCGTTGAAGGATCAATACCAAACTTCTTAATAGAACAAGAAGTTTGGTATTGATCTATTT C.aromaticumMF096912.1 TCGTTGAAGGATCAATACCAAACTTCTTAATAGAACAAGAAGTTTGGTATTGATCTATTT C.aromaticumMG209138.1 TCGTTGAAGGATCAATACCAAACTTCTTAATAGAACAAGAAGTTTGGTATTGATCTATTT C.burmanniHM019384.1 TCGTTGAAGGATCAATACCAAACTTCTTGTTCTATTAAGAAGTTTGGTATTGATCCATTT C.burmanniHM019385.1 TCGTTGAAGGATCAATACCAAACTTCTTGTTCTATTAAGAAGTTTGGTATTGATCCATTT C.burmanniKX546098.1 TCGTTGAAGGATCAATACCAAACTTCTTGTTCTATTAAGAAGTTTGGTATTGATCCATTT C.burmanniKY296407.1 TCGTTGAAGGATCAATACCAAACTTCTTGTTCTATTAAGAAGTTTGGTATTGATCCATTT C.burmanniKY296408.1 TCGTTGAAGGATCAATACCAAACTTCTTGTTCTATTAAGAAGTTTGGTATTGATCCATTT C.burmanniKY296409.1 TCGTTGAAGGATCAATACCAAACTTCTTGTTCTATTAAGAAGTTTGGTATTGATCCATTT C.burmanniKY296410.1 TCGTTGAAGGATCAATACCAAACTTCTTGTTCTATTAAGAAGTTTGGTATTGATCCATTT C.burmanniKY296411.1 TCGTTGAAGGATCAATACCAAACTTCTTGTTCTATTAAGAAGTTTGGTATTGATCCATTT C.burmanniKY296412.1 TCGTTGAAGGATCAATACCAAACTTCTTGTTCTATTAAGAAGTTTGGTATTGATCCATTT C.burmanniKY296413.1 TCGTTGAAGGATCAATACCAAACTTCTTGTTCTATTAAGAAGTTTGGTATTGATCCATTT C.burmanniKY296414.1 TCGTTGAAGGATCAATACCAAACTTCTTGTTCTATTAAGAAGTTTGGTATTGATCCATTT C.loureiroiMF137971.1 TCGTTGAAGGATCAATACCAAACTTCTTGTTCTATTAAGAAGTTTGGTATTGATCCATTT C.verumKF978091.1 TCGTTGAAGGATCAATACCAAACTTCTTGTTCTATTAAGAAGTTTGGTATTGATCCATTT C.verumKF978093.1 TCGTTGAAGGATCAATACCAAACTTCTTGTTCTATTAAGAAGTTTGGTATTGATCCATTT C.verumMH232537.1 TCGTTGAAGGATCAATACCAAACTTCTTGTTCTATTAAGAAGTTTGGTATTGATCCATTT C.verumMH232539.1 TCGTTGAAGGATCAATACCAAACTTCTTGTTCTATTAAGAAGTTTGGTATTGATCCATTT C.verumMH232540.1 TCGTTGAAGGATCAATACCAAACTTCTTGTTCTATTAAGAAGTTTGGTATTGATCCATTT

****************************..*..*..*******************.****

C.aromaticumHM019388.1 GGTTCAGTAGTGTTTTATTCACATAATCGTTTTTCATTTTCATTTCTTTTATTCAACTTA

C.aromaticumHM019389.1 GGTTCAGTAGTGTTTTATTCACATAATCGTTTTTCATTTTCATTTCTTTTATTCAACTTA

C.aromaticumKF978096.1 GGTTCAGTAGTGTTTTATTCACATAATCGTTTTTCATTTTCATTTCTTTTATTCAACTTA

C.aromaticumKF978097.1 GGTTCAGTAGTGTTTTATTCACATAATCGTTTTTCATTTTCATTTCTTTTATTCAACTTA

C.aromaticumKF978098.1 GGTTCAGTAGTGTTTTATTCACATAATCGTTTTTCATTTTCATTTCTTTTATTCAACTTA

C.aromaticumKF978099.1 GGTTCAGTAGTGTTTTATTCACATAATCGTTTTTCATTTTCATTTCTTTTATTCAACTTA

C.aromaticumKX675171.1 GGTTCAGTAGTGTTTTATTCACATAATCGTTTTTCATTTTCATTTCTTTTATTCAACTTA

C.aromaticumKX675172.1 GGTTCAGTAGTGTTTTATTCACATAATCGTTTTTCATTTTCATTTCTTTTATTCAACTTA

C.aromaticumKX675173.1 GGTTCAGTAGTGTTTTATTCACATAATCGTTTTTCATTTTCATTTCTTTTATTCAACTTA

C.aromaticumMF096909.1 GGTTCAGTAGTGTTTTATTCACATAATCGTTTTTCATTTTCATTTCTTTTATTCAACTTA

C.aromaticumMF096910.1 GGTTCAGTAGTGTTTTATTCACATAATCGTTTTTCATTTTCATTTCTTTTATTCAACTTA

C.aromaticumMF096911.1 GGTTCAGTAGTGTTTTATTCACATAATCGTTTTTCATTTTCATTTCTTTTATTCAACTTA

C.aromaticumMF096912.1 GGTTCAGTAGTGTTTTATTCACATAATCGTTTTTCATTTTCATTTCTTTTATTCAACTTA

C.aromaticumMG209138.1 GGTTCAGTAGTGTTTTATTCACATAATCGTTTTTCATTTTCATTTCTTTTATTCAACTTA

C.burmanniHM019384.1 GGTTCAGTAGTGTTTTATTCACATAATCGTTTTTCATTTTCATTTCTTTTATTCAACTTA

C.burmanniHM019385.1 GGTTCAGTAGTGTTTTATTCACATAATCGTTTTTCATTTTCATTTCTTTTATTCAACTTA

C.burmanniKX546098.1 GGTTCAGTAGTGTTTTATTCACATAATCGTTTTTCATTTTCATTTCTTTTATTCAACTTA

C.burmanniKY296407.1 GGTTCAGTAGTGTTTTATTCACATAATCGTTTTTCATTTTCATTTCTTTTATTCAACTTA

C.burmanniKY296408.1 GGTTCAGTAGTGTTTTATTCACATAATCGTTTTTCATTTTCATTTCTTTTATTCAACTTA

C.burmanniKY296409.1 GGTTCAGTAGTGTTTTATTCACATAATCGTTTTTCATTTTCATTTCTTTTATTCAACTTA

C.burmanniKY296410.1 GGTTCAGTAGTGTTTTATTCACATAATCGTTTTTCATTTTCATTTCTTTTATTCAACTTA

C.burmanniKY296411.1 GGTTCAGTAGTGTTTTATTCACATAATCGTTTTTCATTTTCATTTCTTTTATTCAACTTA

C.burmanniKY296412.1 GGTTCAGTAGTGTTTTATTCACATAATCGTTTTTCATTTTCATTTCTTTTATTCAACTTA

C.burmanniKY296413.1 GGTTCAGTAGTGTTTTATTCACATAATCGTTTTTCATTTTCATTTCTTTTATTCAACTTA

C.burmanniKY296414.1 GGTTCAGTAGTGTTTTATTCACATAATCGTTTTTCATTTTCATTTCTTTTATTCAACTTA

C.loureiroiMF137971.1 GGTTCAGTAGTGTTTTATTCACATAATCGTTTTTCATTTTCATTTCTTTTATTCAACTTA

C.verumKF978091.1 GGTTAAGTAGTGTTTTATTCACATAATCGTTTTTCATTTTAATTTCTTTTATTCAACTTA

C.verumKF978093.1 GGTTAAGTAGTGTTTTATTCACATAATCGTTTTTCATTTTAATTTCTTTTATTCAACTTA

C.verumMH232537.1 GGTTAAGTAGTGTTTTATTCACATAATCGTTTTTCATTTTAATTTCTTTTATTCAACTTA

C.verumMH232539.1 GGTTAAGTAGTGTTTTATTCACATAATCGTTTTTCATTTTAATTTCTTTTATTCAACTTA

C.verumMH232540.1 GGTTAAGTAGTGTTTTATTCACATAATCGTTTTTCATTTTAATTTCTTTTATTCAACTTA

****.***********************************.*******************

C.aromaticumHM019388.1 TGAAAACCGCTGGTTATTTCATGATCGAATATCGTAGTTTCTTCTGTACCAACCTGCATT

C.aromaticumHM019389.1 TGAAAACCGCTGGTTATTTCATGATCGAATATCGTAGTTTCTTCTGTACCAACCTGCATT

C.aromaticumKF978096.1 TGAAAACCGCTGGTTATTTCATGATCGAATATCGTAGTTTCTTCTGTACCAACCTGCATT

C.aromaticumKF978097.1 TGAAAACCGCTGGTTATTTCATGATCGAATATCGTAGTTTCTTCTGTACCAACCTGCATT

C.aromaticumKF978098.1 TGAAAACCGCTGGTTATTTCATGATCGAATATCGTAGTTTCTTCTGTACCAACCTGCATT

C.aromaticumKF978099.1 TGAAAACCGCTGGTTATTTCATGATCGAATATCGTAGTTTCTTCTGTACCAACCTGCATT

C.aromaticumKX675171.1 TGAAAACCGCTGGTTATTTCATGATCGAATATCGTAGTTTCTTCTGTACCAACCTGCATT

C.aromaticumKX675172.1 TGAAAACCGCTGGTTATTTCATGATCGAATATCGTAGTTTCTTCTGTACCAACCTGCATT

C.aromaticumKX675173.1 TGAAAACCGCTGGTTATTTCATGATCGAATATCGTAGTTTCTTCTGTACCAACCTGCATT

C.aromaticumMF096909.1 TGAAAACCGCTGGTTATTTCATGATCGAATATCGTAGTTTCTTCTGTACCAACCTGCATT

C.aromaticumMF096910.1 TGAAAACCGCTGGTTATTTCATGATCGAATATCGTAGTTTCTTCTGTACCAACCTGCATT

C.aromaticumMF096911.1 TGAAAACCGCTGGTTATTTCATGATCGAATATCGTAGTTTCTTCTGTACCAACCTGCATT

C.aromaticumMF096912.1 TGAAAACCGCTGGTTATTTCATGATCGAATATCGTAGTTTCTTCTGTACCAACCTGCATT

C.aromaticumMG209138.1 TGAAAACCGCTGGTTATTTCATGATCGAATATCGTAGTTTCTTCTGTACCAACCTGCATT

C.burmanniHM019384.1 TGAAAACCGCTGGTTATTTCATGATCGAATATCGTAGTTTCTTCTGTACCAACCTGCATT

C.burmanniHM019385.1 TGAAAACCGCTGGTTATTTCATGATCGAATATCGTAGTTTCTTCTGTACCAACCTGCATT

C.burmanniKX546098.1 TGAAAACCGCTGGTTATTTCATGATCGAATATCGTAGTTTCTTCTGTACCAACCTGCATT

C.burmanniKY296407.1 TGAAAACCGCTGGTTATTTCATGATCGAATATCGTAGTTTCTTCTGTACCAACCTGCATT

C.burmanniKY296408.1 TGAAAACCGCTGGTTATTTCATGATCGAATATCGTAGTTTCTTCTGTACCAACCTGCATT

C.burmanniKY296409.1 TGAAAACCGCTGGTTATTTCATGATCGAATATCGTAGTTTCTTCTGTACCAACCTGCATT

C.burmanniKY296410.1 TGAAAACCGCTGGTTATTTCATGATCGAATATCGTAGTTTCTTCTGTACCAACCTGCATT

C.burmanniKY296411.1 TGAAAACCGCTGGTTATTTCATGATCGAATATCGTAGTTTCTTCTGTACCAACCTGCATT

C.burmanniKY296412.1 TGAAAACCGCTGGTTATTTCATGATCGAATATCGTAGTTTCTTCTGTACCAACCTGCATT

C.burmanniKY296413.1 TGAAAACCGCTGGTTATTTCATGATCGAATATCGTAGTTTCTTCTGTACCAACCTGCATT

C.burmanniKY296414.1 TGAAAACCGCTGGTTATTTCATGATCGAATATCGTAGTTTCTTCTGTACCAACCTGCATT

C.loureiroiMF137971.1 TGAAAACCGCTGGTTATTTCATGATCGAATATCGTAGTTTCTTCTGTACCAACCTGCATT

C.verumKF978091.1 TGAAAACCGCTGGTTATTTCATGATCGAATATCGTAGTTTCTTCTGTACCAACCTGCATT

C.verumKF978093.1 TGAAAACCGCTGGTTATTTCATGATCGAATATCGTAGTTTCTTCTGTACCAACCTGCATT

C.verumMH232537.1 TGAAAACCGCTGGTTATTTCATGATCGAATATCGTAGTTTCTTCTGTACCAACCTGCATT

C.verumMH232539.1 TGAAAACCGCTGGTTATTTCATGATCGAATATCGTAGTTTCTTCTGTACCAACCTGCATT

C.verumMH232540.1 TGAAAACCGCTGGTTATTTCATGATCGAATATCGTAGTTTCTTCTGTACCAACCTGCATT

************************************************************

C.aromaticumHM019388.1 TTATATACTCTTATTCTTCAAAATAATTTGATTTTTTGAAAAAATCAAAGCATTTTTCTT C.aromaticumHM019389.1 TTATATACTCTTATTCTTCAAAATAATTTGATTTTTTGAAAAAATCAAAGCATTTTTCTT C.aromaticumKF978096.1 TTATATACTCTTATTCTTCAAAATAATTTGATTTTTTGAAAAAATCAAAGCATTTTTCTT C.aromaticumKF978097.1 TTATATACTCTTATTCTTCAAAATAATTTGATTTTTTGAAAAAATCAAAGCATTTTTCTT C.aromaticumKF978098.1 TTATATACTCTTATTCTTCAAAATAATTTGATTTTTTGAAAAAATCAAAGCATTTTTCTT C.aromaticumKF978099.1 TTATATACTCTTATTCTTCAAAATAATTTGATTTTTTGAAAAAATCAAAGCATTTTTCTT C.aromaticumKX675171.1 TTATATACTCTTATTCTTCAAAATAATTTGATTTTTTGAAAAAATCAAAGCATTTTTCTT C.aromaticumKX675172.1 TTATATACTCTTATTCTTCAAAATAATTTGATTTTTTGAAAAAATCAAAGCATTTTTCTT C.aromaticumKX675173.1 TTATATACTCTTATTCTTCAAAATAATTTGATTTTTTGAAAAAATCAAAGCATTTTTCTT C.aromaticumMF096909.1 TTATATACTCTTATTCTTCAAAATAATTTGATTTTTTGAAAAAATCAAAGCATTTTTCTT C.aromaticumMF096910.1 TTATATACTCTTATTCTTCAAAATAATTTGATTTTTTGAAAAAATCAAAGCATTTTTCTT C.aromaticumMF096911.1 TTATATACTCTTATTCTTCAAAATAATTTGATTTTTTGAAAAAATCAAAGCATTTTTCTT C.aromaticumMF096912.1 TTATATACTCTTATTCTTCAAAATAATTTGATTTTTTGAAAAAATCAAAGCATTTTTCTT C.aromaticumMG209138.1 TTATATACTCTTATTCTTCAAAATAATTTGATTTTTTGAAAAAATCAAAGCATTTTTCTT C.burmanniHM019384.1 TTATATACTCTTATTCTTCAAAATAATTTTATTTTTTGAAAAAATCAAAGCATTTTTCTT C.burmanniHM019385.1 TTATATACTCTTATTCTTCAAAATAATTTTATTTTTTGAAAAAATCAAAGCATTTTTCTT C.burmanniKX546098.1 TTATATACTCTTATTCTTCAAAATAATTTTATTTTTTGAAAAAATCAAAGCATTTTTCTT C.burmanniKY296407.1 TTATATACTCTTATTCTTCAAAATAATTTTATTTTTTGAAAAAATCAAAGCATTTTTCTT C.burmanniKY296408.1 TTATATACTCTTATTCTTCAAAATAATTTTATTTTTTGAAAAAATCAAAGCATTTTTCTT C.burmanniKY296409.1 TTATATACTCTTATTCTTCAAAATAATTTTATTTTTTGAAAAAATCAAAGCATTTTTCTT C.burmanniKY296410.1 TTATATACTCTTATTCTTCAAAATAATTTTATTTTTTGAAAAAATCAAAGCATTTTTCTT C.burmanniKY296411.1 TTATATACTCTTATTCTTCAAAATAATTTTATTTTTTGAAAAAATCAAAGCATTTTTCTT C.burmanniKY296412.1 TTATATACTCTTATTCTTCAAAATAATTTTATTTTTTGAAAAAATCAAAGCATTTTTCTT C.burmanniKY296413.1 TTATATACTCTTATTCTTCAAAATAATTTTATTTTTTGAAAAAATCAAAGCATTTTTCTT C.burmanniKY296414.1 TTATATACTCTTATTCTTCAAAATAATTTTATTTTTTGAAAAAATCAAAGCATTTTTCTT C.loureiroiMF137971.1 TTATATCCTCTTATTCTTCAAAATAATTTGATTTTTTGAAAAAATCAAAGCATTTTTCTT C.verumKF978091.1 TTATATACTCTTATTCTTCAAAATAATTTGATTTTAGGAAAAAATCAAAGCATTTTTCTT C.verumKF978093.1 TTATATACTCTTATTCTTCAAAATAATTTGATTTTAGGAAAAAATCAAAGCATTTTTCTT C.verumMH232537.1 TTATATACTCTTATTCTTCAAAATAATTTGATTTTAGGAAAAAATCAAAGCATTTTTCTT C.verumMH232539.1 TTATATACTCTTATTCTTCAAAATAATTTGATTTTAGGAAAAAATCAAAGCATTTTTCTT C.verumMH232540.1 TTATATACTCTTATTCTTCAAAATAATTTGATTTTAGGAAAAAATCAAAGCATTTTTCTT

******.**********************.*****..***********************

**B**

HM019384.1Cinnamomum burmanni GTTCCATCTACAAACGGATAATACTTTGGTATTAGTGTATACGAGTCGTTGAAGGATCAA KF978091.1Cinnamomum verum GTTCCATCTACAAACGGATAATACTTTGGTATTAGTGTATACGAGTCGTTGAAGGATCAA KF978096.1Cinnamomum aromaticum GTTCCATCTACAAACGGATAATACTTTGGTATTAGTGTATACGAGTCGTTGAAGGATCAA MF137971.1Cinnamomum loureiroi GTTCCATCTACAAACGGATAATACTTTGGTATTAGTGTATACGAGTCGTTGAAGGATCAA

***********************************************************

HM019384.1Cinnamomum burmanni TACCAAACTTCTTGTTCTATTAAGAAGTTTGGTATTGATCCATTTGGTTCAGTAGTGTTT KF978091.1Cinnamomum verum TACCAAACTTCTTGTTCTATTAAGAAGTTTGGTATTGATCCATTTGGTTAAGTAGTGTTT KF978096.1Cinnamomum aromaticum TACCAAACTTCTTAATAGAACAAGAAGTTTGGTATTGATCTATTTGGTTCAGTAGTGTTT MF137971.1Cinnamomum loureiroi TACCAAACTTCTTGTTCTATTAAGAAGTTTGGTATTGATCCATTTGGTTCAGTAGTGTTT

*************..*..*..*******************.********.*********

HM019384.1Cinnamomum burmanni TATTCACATAATCGTTTTTCATTTTCATTTCTTTTATTCAACTTATGAAAACCGCTGGTT KF978091.1Cinnamomum verum TATTCACATAATCGTTTTTCATTTTAATTTCTTTTATTCAACTTATGAAAACCGCTGGTT KF978096.1Cinnamomum aromaticum TATTCACATAATCGTTTTTCATTTTCATTTCTTTTATTCAACTTATGAAAACCGCTGGTT MF137971.1Cinnamomum loureiroi TATTCACATAATCGTTTTTCATTTTCATTTCTTTTATTCAACTTATGAAAACCGCTGGTT

*************************.*********************************

HM019384.1Cinnamomum burmanni ATTTCATGATCGAATATCGTAGTTTCTTCTGTACCAACCTGCATTTTATATACTCTTATT KF978091.1Cinnamomum verum ATTTCATGATCGAATATCGTAGTTTCTTCTGTACCAACCTGCATTTTATATACTCTTATT KF978096.1Cinnamomum aromaticum ATTTCATGATCGAATATCGTAGTTTCTTCTGTACCAACCTGCATTTTATATACTCTTATT MF137971.1Cinnamomum loureiroi ATTTCATGATCGAATATCGTAGTTTCTTCTGTACCAACCTGCATTTTATATCCTCTTATT

***************************************************.*******

HM019384.1Cinnamomum burmanni CTTCAAAATAATTTTATTTTTTGAAAAAATCAAAGCATTTTTCTTTTTTTTTTTTTT--- KF978091.1Cinnamomum verum CTTCAAAATAATTTGATTTTAGGAAAAAATCAAAGCATTTTTCTTTTTTTTTTTTTTT-- KF978096.1Cinnamomum aromaticum CTTCAAAATAATTTGATTTTTTGAAAAAATCAAAGCATTTTTCTTTTTTTTTTTTTTTT- MF137971.1Cinnamomum loureiroi CTTCAAAATAATTTGATTTTTTGAAAAAATCAAAGCATTTTTCTTTTTTTTTTTTTTTTT

**************.*****..***********************************...

HM019384.1Cinnamomum burmanni ACGTACAACATTTTGTTTTATGTACAATATCTGTATTTCAGCAGGAAGGAGAGTGAAGTA KF978091.1Cinnamomum verum ACGTACAACATTTTGTTTTATGTACAATATCTGTATTTCAGCAGGAAGGAGAGTGAAGTA KF978096.1Cinnamomum aromaticum ACGTACAACATTTTGTTTTATGTACAATATCTGTATTTCAGCAGGAAGGAGAGTGAAGTA MF137971.1Cinnamomum loureiroi ACGTACAACATTTTGTTTTATGTACAATATCTGTATTTCAGCAGGAAGGAGAGTGAAGTA

***********************************************************

HM019384.1Cinnamomum burmanni ATAAAGAC

KF978091.1Cinnamomum verum ATAAAGAC

KF978096.1Cinnamomum aromaticum ATAAAGAC

MF137971.1Cinnamomumloureiroi ATAAAGAC

********

**S2 Fig.** **Melting curve profiles of amplicons obtained from the novel *AP*-*trn*H-*psb*A marker and *afl*R marker (multiplex)** (A) Normalized melting curves, (B) Difference melting curves of the *C. verum, C. aromaticum, C. burmanni, C. loureiroi,* and *A. flavus*.

**
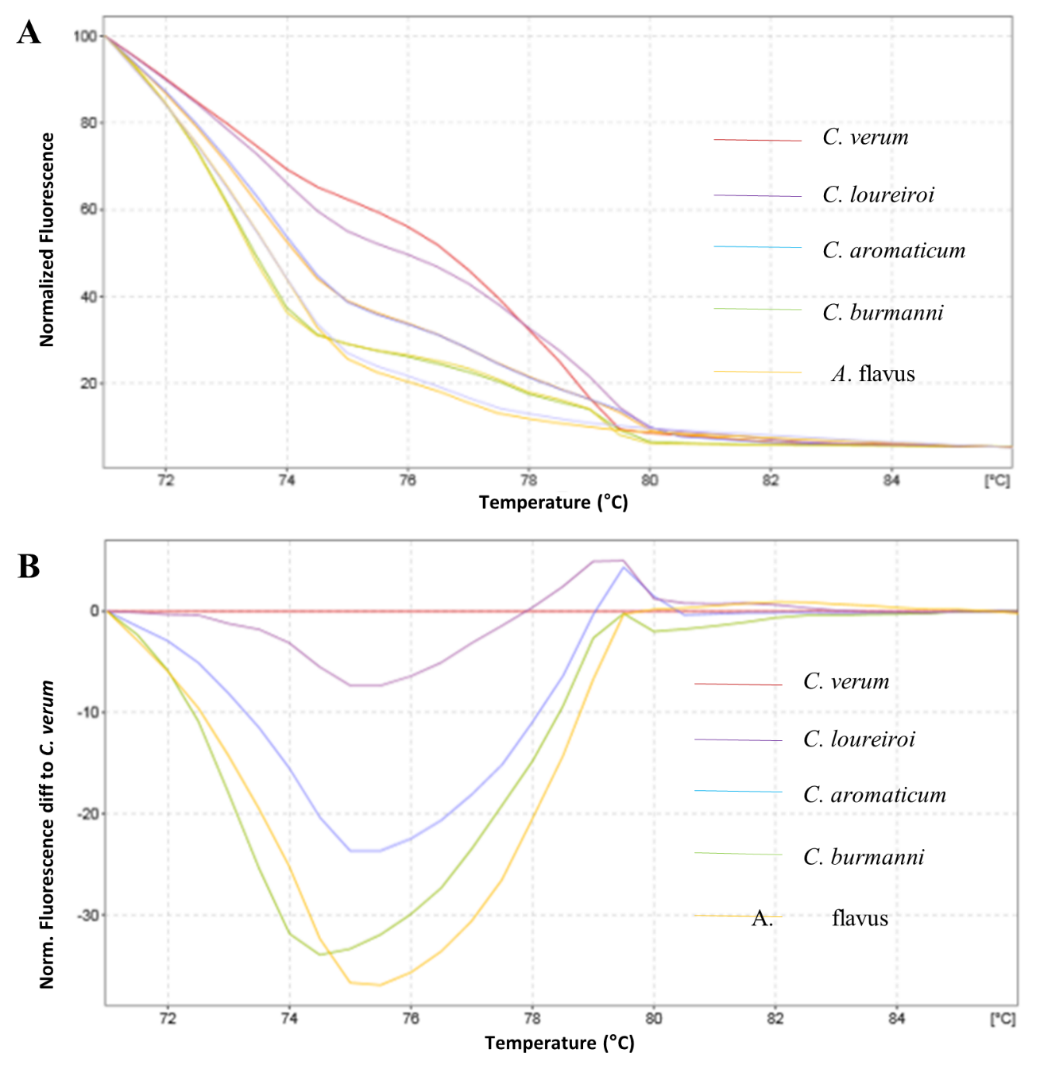
**

**S3 Fig.** **Sequence alignments of *C. verum* in the *trn*H-*psb*A barcode region**(A) Sequences of samples collected from agricultural fields and (B) *C. verum* sequences retrieved from GenBank (NCBI).

**A**

Sample1 ACTTTGGTATTAGTGTATACGAGTCGTTGAAGGATCAATACCAAACTTCTTGTTCTATTA

Sample2 ACTTTGGTATTAGTGTATACGAGTCGTTGAAGGATCAATACCAAACTTCTTGTTCTATTA

Sample3 ACTTTGGTATTAGTGTATACGAGTCGTTGAAGGATCAATACCAAACTTCTTGTTCTATTA

Sample4 ACTTTGGTATTAGTGTATACGAGTCGTTGAAGGATCAATACCAAACTTCTTGTTCTATTA

Sample5 ACTTTGGTATTAGTGTATACGAGTCGTTGAAGGATCAATACCAAACTTCTTGTTCTATTA

Sample6 ACTTTGGTATTAGTGTATACGAGTCGTTGAAGGATCAATACCAAACTTCTTGTTCTATTA

Sample7 ACTTTGGTATTAGTGTATACGAGTCGTTGAAGGATCAATACCAAACTTCTTGTTCTATTA

Sample8 ACTTTGGTATTAGTGTATACGAGTCGTTGAAGGATCAATACCAAACTTCTTGTTCTATTA

Sample9 ACTTTGGTATTAGTGTATACGAGTCGTTGAAGGATCAATACCAAACTTCTTAATAGAACA

Sample10 ACTTTGGTATTAGTGTATACGAGTCGTTGAAGGATCAATACCAAACTTCTTAATAGAACA

Sample11 ACTTTGGTATTAGTGTATACGAGTCGTTGAAGGATCAATACCAAACTTCTTAATAGAACA

*************************************************** * * *

Sample1 AGAAGTTTGGTATTGATCCATTTGGTTAAGTAGTGTTTTATTCACATAATCGTTTTTCAT

Sample2 AGAAGTTTGGTATTGATCCATTTGGTTAAGTAGTGTTTTATTCACATAATCGTTTTTCAT

Sample3 AGAAGTTTGGTATTGATCCATTTGGTTAAGTAGTGTTTTATTCACATAATCGTTTTTCAT

Sample4 AGAAGTTTGGTATTGATCCATTTGGTTAAGTAGTGTTTTATTCACATAATCGTTTTTCAT

Sample5 AGAAGTTTGGTATTGATCCATTTGGTTAAGTAGTGTTTTATTCACATAATCGTTTTTCAT

Sample6 AGAAGTTTGGTATTGATCCATTTGGTTAAGTAGTGTTTTATTCACATAATCGTTTTTCAT

Sample7 AGAAGTTTGGTATTGATCCATTTGGTTAAGTAGTGTTTTATTCACATAATCGTTTTTCAT

Sample8 AGAAGTTTGGTATTGATCCATTTGGTTAAGTAGTGTTTTATTCACATAATCGTTTTTCAT

Sample9 AGAAGTTTGGTATTGATCCATTTGGTTAAGTAGTGTTTTATTCACATAATCGTTTTTCAT

Sample10 AGAAGTTTGGTATTGATCCATTTGGTTAAGTAGTGTTTTATTCACATAATCGTTTTTCAT

Sample11 AGAAGTTTGGTATTGATCCATTTGGTTAAGTAGTGTTTTATTCACATAATCGTTTTTCAT

************************************************************

**B**

KY966337.1 ACTTTGGTATTAGTGTATACGAGTCGTTGAAGGATCAATACCAAACTTCTTGTTCTATTA

KY966336.1 ACTTTGGTATTAGTGTATACGAGTCGTTGAAGGATCAATACCAAACTTCTTGTTCTATTA

KY966338.1 ACTTTGGTATTAGTGTATACGAGTCGTTGAAGGATCAATACCAAACTTCTTGTTCTATTA

KY966339.1 ACTTTGGTATTAGTGTATACGAGTCGTTGAAGGATCAATACCAAACTTCTTGTTCTATTA

KY296447.1 ACTTTGGTATTAGTGTATACGAGTCGTTGAAGGATCAATACCAAACTTCTTGTTCTATTA

MH069893.1 ACTTTGGTATTAGTGTATACGAGTCGTTGAAGGATCAATACCAAACTTCTTGTTCTATTA

MH232537.1 ACTTTGGTATTAGTGTATACGAGTCGTTGAAGGATCAATACCAAACTTCTTGTTCTATTA

MH232539.1 ACTTTGGTATTAGTGTATACGAGTCGTTGAAGGATCAATACCAAACTTCTTGTTCTATTA

MH232540.1 ACTTTGGTATTAGTGTATACGAGTCGTTGAAGGATCAATACCAAACTTCTTGTTCTATTA

KF978091.1 ACTTTGGTATTAGTGTATACGAGTCGTTGAAGGATCAATACCAAACTTCTTGTTCTATTA

KF978093.1 ACTTTGGTATTAGTGTATACGAGTCGTTGAAGGATCAATACCAAACTTCTTGTTCTATTA

MK090019.1 ACTTTGGTATTAGTGTATACGAGTCGTTGAAGGATCAATACCAAACTTCTTAATAGAACA

KF978095.1 ACTTTGGTATTAGTGTATACGAGTCGTTGAAGGATCAATACCAAACTTCTTAATAGAACA

MH232541.1 ACTTTGGTATTAGTGTATACGAGTCGTTGAAGGATCAATACCAAACTTCTTAATAGAACA

MH232538.1 ACTTTGGTATTAGTGTATACGAGTCGTTGAAGGATCAATACCAAACTTCTTAATAGAACA

MH069894.1 ACTTTGGTATTAGTGTATACGAGTCGTTGAAGGATCAATACCAAACTTCTTAATAGAACA

KY966341.1 ACTTTGGTATTAGTGTATACGAGTCGTTGAAGGATCAATACCAAACTTCTTAATAGAACA

*************************************************** * * *

KY966337.1 AGAAGTTTGGTATTGATCCATTTGGTTAAGTAGTGTTTTATTCACATAATCGTTTTTCAT

KY966336.1 AGAAGTTTGGTATTGATCCATTTGGTTAAGTAGTGTTTTATTCACATAATCGTTTTTCAT

KY966338.1 AGAAGTTTGGTATTGATCCATTTGGTTAAGTAGTGTTTTATTCACATAATCGTTTTTCAT

KY966339.1 AGAAGTTTGGTATTGATCCATTTGGTTAAGTAGTGTTTTATTCACATAATCGTTTTTCAT

KY296447.1 AGAAGTTTGGTATTGATCCATTTGGTTAAGTAGTGTTTTATTCACATAATCGTTTTTCAT

MH069893.1 AGAAGTTTGGTATTGATCCATTTGGTTAAGTAGTGTTTTATTCACATAATCGTTTTTCAT

MH232537.1 AGAAGTTTGGTATTGATCCATTTGGTTAAGTAGTGTTTTATTCACATAATCGTTTTTCAT

MH232539.1 AGAAGTTTGGTATTGATCCATTTGGTTAAGTAGTGTTTTATTCACATAATCGTTTTTCAT

MH232540.1 AGAAGTTTGGTATTGATCCATTTGGTTAAGTAGTGTTTTATTCACATAATCGTTTTTCAT

KF978091.1 AGAAGTTTGGTATTGATCCATTTGGTTAAGTAGTGTTTTATTCACATAATCGTTTTTCAT

KF978093.1 AGAAGTTTGGTATTGATCCATTTGGTTAAGTAGTGTTTTATTCACATAATCGTTTTTCAT

MK090019.1 AGAAGTTTGGTATTGATCCATTTGGTTAAGTAGTGTTTTATTCACATAATCGTTTTTCAT

KF978095.1 AGAAGTTTGGTATTGATCCATTTGGTTAAGTAGTGTTTTATTCACATAATCGTTTTTCAT

MH232541.1 AGAAGTTTGGTATTGATCCATTTGGTTAAGTAGTGTTTTATTCACATAATCGTTTTTCAT

MH232538.1 AGAAGTTTGGTATTGATCCATTTGGTTAAGTAGTGTTTTATTCACATAATCGTTTTTCAT

MH069894.1 AGAAGTTTGGTATTGATCCATTTGGTTAAGTAGTGTTTTATTCACATAATCGTTTTTCAT

KY966341.1 AGAAGTTTGGTATTGATCCATTTGGTTAAGTAGTGTTTTATTCACATAATCGTTTTTCAT

************************************************************

**S4 Fig. Difference melting curves of the *C. verum*, *C. aromaticum*, *C. burmanni*, *C. loureiroi*, and commercial products were derived from amplicons obtained using the novel *AP*-*trn*H-*psb*A marker.**

**
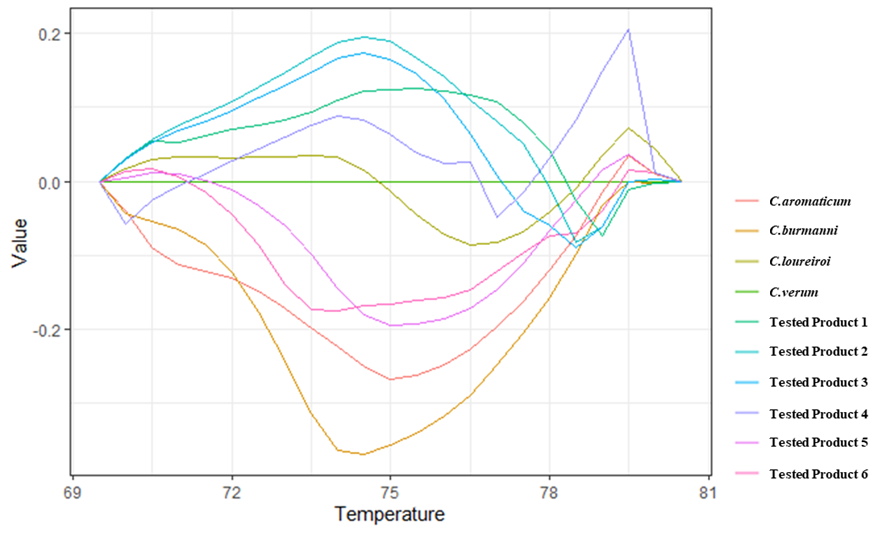
**

**S1 Table.** **DNA sequences corresponding to the *rbc*L, *trn*H-*psb*A, *mat*K, ITS2, *trn*L, and *trn*L-*trn*F regions of *Cinnamomum* species were retrieved from GenBank (NCBI), with accession numbers documented for each species.**

| **Species** | **Barcode region** | | | | | |
| --- | --- | --- | --- | --- | --- | --- |
|  | ***rbc*L** | ***psb*A-*trn*H** | ***mat*K** | **ITS2** | ***trn*L** | ***trn*L-*trn*F** |
| ***C. verum*** | MK090207.1  MH069761.1  JX414039.1  JX843243.1  KF744226.1  KF744227.1  KF744228.1  KF744229.1  KF744230.1 | KF978091.1  KF978093.1  MH232537.1  MH232539.1  MH232540.1 | MG280937.1  MK089931.1  KP318139.1  KP318142.1  JX414035.1  JX495693.1  MF962747.1  MF962748.1  MF991903.1 | GQ255634.1  FJ357245.1 | AB040086.1  AB054242.1  AB054243.1 | AB040076.1 |
| ***C. aromaticum*** | KP094936.1  KJ439992.1  HM019458.1  HM019459.1  KF878109.1  KF878110.1  KF878111.1  KF878112.1  KF878113.1 | MG209138.1 HM019388.1 HM019389.1 KX675171.1 KX675172.1 KX675173.1  MF096909.1 MF096910.1 MF096911.1 MF096912.1 KF978096.1 KF978097.1 KF978098.1 KF978099.1 | MF627715.1  MF627716.1  MF627717.1  MF627718.1  MF627719.1  HM019318.1  HM019319.1  KP093991.1  GQ434289.1 | JF755931.1 JF755932.1 JF755933.1  MF096125.1 MF096126.1 MF096127.1 MF096128.1 | AB040085.1  AB054236.1  AB054237.1  AB054238.1  AB054239.1  AB054240.1  AB054241.1 | AB040075.1  AB054228.1  AB054229.1  AB054230.1  AB054231.1  AB054232.1  AB054233.1 |
| ***C. loureiroi*** | JX843241.1 | MF137971.1 | JQ435500.1 | GQ255632.1 |  |  |
| ***C. burmanni*** | KP094209.1  KP094210.1  HM019454.1  HM019455.1  KX546827.1  KX546828.1  MK090284.1 | HM019384.1 HM019385.1 KX546098.1 KY296407.1 KY296408.1 KY296409.1 KY296410.1 KY296411.1 KY296412.1 KY296413.1 KY296414.1 | KF740402.1  KX545695.1  KX545696.1  HM019314.1  HM019315.1  GQ434290.1  KP093299.1  KP093300.1  MK089981.1 | GQ255636.1 GQ255637.1 GQ255638.1  FJ357247.1  FJ357248.1 | DQ822590.1  DQ822591.1 | AB040077.1 |

**S2 Table.** **Comparison of nucleotide variation among *Cinnamomum* species** A. Number of SNPs specific to each gene region which can be used to distinguish up to species level, B. position of nucleotide variation of *rbc*L, and *trn*H-*psb*A, *trn*L, *trn*L-*trn*F *mat*K, and ITS2 regions of *Cinnamomum* species.

**A**

| **Species** | **Barcode region** | | | |
| --- | --- | --- | --- | --- |
|  | ***rbc*L** | *trn*H*-psb*A | ***mat*K** | **ITS2** |
| *C. verum* | 0 | 4 | 0 | 21 |
| *C. aromaticum* | 1 | 7 | 0 | 27 |
| *C. burmanni* | 0 | 1 | 0 | 4 |
| *C. loureiroi* | 14 | 1 | 14 | 7 |

| **Species** | ***rbc*L** | | | | | | | | | | | | | | | | |
| --- | --- | --- | --- | --- | --- | --- | --- | --- | --- | --- | --- | --- | --- | --- | --- | --- | --- |
|  | 16 | 17 | 231 | 350 | 358 | 359 | 362 | 386 | 393 | 400 | 402 | 409 | 415 | 419 | 442 | 444 | 447 |
| *C. verum* | A | G | T | A | A | C | T | A | G | A | A | A | G | G | A | A | A |
| *C. aromaticum* | G | A | C | A | A | C | T | A | G | A | A | A | G | G | A | A | A |
| *C. burmanni* | G | A | T | A | A | C | T | A | G | A | A | A | G | G | A | A | A |
| *C. loureiroi* | A | G | T | T | C | T | C | C | A | T | T | C | T | C | T | T | C |

**B**

| **Species** | ***trn*H*-psb*A** | | | | | | | | | | | | | ***trn*L** | | | ***trn*L-*trn*F** |
| --- | --- | --- | --- | --- | --- | --- | --- | --- | --- | --- | --- | --- | --- | --- | --- | --- | --- |
|  | 35 | 36 | 38 | 39 | 41 | 42 | 62 | 71 | 107 | 193 | 216 | 222 | 223 | 244 | 254 | 279 | 80 |
| *C. verum* | G | T | C | T | T | T | C | **A** | **A** | A | G | **A** | **G** | G | T | G | C |
| *C. aromaticum* | **A** | **A** | **A** | **G** | **A** | **C** | **T** | C | C | A | G | T | T | G | G | T | C |
| *C. burmanni* | G | T | C | T | T | T | C | C | C | A | **T** | T | T | T | T | T | T |
| *C. loureiroi* | G | T | C | T | T | T | C | C | C | **C** | G | T | T | - | - | - | - |

| **Species** | ***mat*K** | | | | | | | | | | | | | | |
| --- | --- | --- | --- | --- | --- | --- | --- | --- | --- | --- | --- | --- | --- | --- | --- |
|  | 264 | 282 | 288 | 298 | 309 | 378 | 399 | 408 | 431 | 473 | 494 | 498 | 499 | 500 | 509 |
| *C. verum* | T | A | T | A | T | C | A | G | A | C | A | A | A | A | A |
| *C. aromaticum* | T | A | T | A | T | G | A | G | A | C | A | A | A | A | A |
| *C. burmanni* | T | A | T | A | T | G | A | G | A | C | A | A | A | A | A |
| *C. loureiroi* | C | C | C | G | A | C | C | A | T | A | C | C | C | C | G |

| **Species** | **ITS2** | | | | | | | | | | | | | | | | | | | | |
| --- | --- | --- | --- | --- | --- | --- | --- | --- | --- | --- | --- | --- | --- | --- | --- | --- | --- | --- | --- | --- | --- |
|  | 2 | 5 | 9 | 10 | 11 | 16 | 17 | 24 | 27 | 29 | 30 | 32 | 35 | 37 | 48 | 62 | 63 | 74 | 75 | 76 | 77 |
| *C. verum* | G | G | A | - | T | T | C | C | G | T | C | T | - | A | A | T | C | A | T | G | C |
| *C. aromaticum* | A | G | A | C | T | G | T | T | G | T | A | T | G | G | C | C | A | G | C | A | T |
| *C. burmanni* | G | G | A | A | T | T | C | C | A | C | G | T | G | G | A | C | C | A | C | A | C |
| *C. loureiroi* | G | T | G | A | A | C | C | C | G | T | G | C | G | G | A | C | C | A | C | A | C |

| **Species** | **ITS2** | | | | | | | | | | | | | | | | | |  |
| --- | --- | --- | --- | --- | --- | --- | --- | --- | --- | --- | --- | --- | --- | --- | --- | --- | --- | --- | --- |
|  | 78 | 80 | 89 | 90 | 92 | 93 | 100 | 104 | 109 | 111 | 115 | 121 | 122 | 124 | 132 | 135 | 138 | 139 | |
| *C. verum* | A | C | A | A | - | - | C | T | G | T | T | C | A | A | T | C | C | A | |
| *C. aromaticum* | G | T | G | T | - | - | T | T | A | A | C | T | T | C | T | T | T | G | |
| *C. burmanni* | G | C | G | T | G | T | C | C | G | C | C | T | C | C | A | C | C | G | |
| *C. loureiroi* | G | C | G | T | - | - | C | C | G | C | C | T | C | C | A | C | C | G | |

| **Species** | **ITS2** | | | | | | | | | | | | | | | | |
| --- | --- | --- | --- | --- | --- | --- | --- | --- | --- | --- | --- | --- | --- | --- | --- | --- | --- |
|  | 144 | 148 | 150 | 153 | 162 | 164 | 168 | 170 | 171 | 172 | 174 | 177 | 178 | 179 | 183 | 188 | 192 |
| *C. verum* | G | C | - | C | T | A | G | C | C | A | G | G | G | C | T | G | C |
| *C. aromaticum* | A | C | - | A | A | G | T | T | T | G | A | C | A | T | C | A | A |
| *C. burmanni* | G | C | - | C | A | A | G | C | C | G | A | C | G | C | C | G | C |
| *C. loureiroi* | G | A | G | C | A | A | G | C | C | G | A | C | G | C | C | G | C |

**S3 Table.**  **Details of the samples that were used for the study.**

| No | Species | Number of samples | Source | Sample validation |
| --- | --- | --- | --- | --- |
| 01 | *C. verum* | 05 | NCBI  KF978091.1, KF978093.1, MH232537.1, MH232539.1, MH232540.1 |  |
|  |  | 09 | Fresh leaf samples | Sanger sequencing and validation |
|  |  | 05 | Commercial product | Sanger sequencing and validation |
| 02 | *C.* *aromaticum* | 14 | NCBI  MG209138.1, HM019388.1, HM019389.1, KX675171.1, KX675172.1, KX675173.1, MF096909.1, MF096910.1, MF096911.1, MF096912.1, KF978096.1, KF978097.1, KF978098.1, KF978099.1 |  |
|  |  | 03 | Commercial product | Sanger sequencing and validation |
| 03 | *C. burmanni* | 11 | NCBI  HM019384.1, HM019385.1, KX546098.1, KY296407.1, KY296408.1, KY296409.1, KY296410.1, KY296411.1, KY296412.1, KY296413.1, KY296414.1 |  |
|  |  | 03 | Commercial product | Sanger sequencing and validation |
| 04 | *C.* *loureiroi* | 01 | NCBI  MF137971.1 |  |
|  |  | 02 | Commercial product | Sanger sequencing and validation |
